# Supplementary material for: Use of corticoids and non-steroidal anti-inflammatories in the treatment of rheumatoid arthritis: Systematic review and network meta-analysis
Source: PLoS One. 2021 Apr 7;16(4):e0248866. doi: 10.1371/journal.pone.0248866 (PMC8026036; doi:10.1371/journal.pone.0248866)
Supplement: S1 File — (DOCX) [file pone.0248866.s002.docx]

S1 File. Search strategies for different databases.

**COCHRANE CENTRAL: 1,428**

(Arthritis, Rheumatoid) AND (Anti Inflammatory Agents, Non Steroidal OR Glucocorticoid) AND (randomized controlled trial)

**MEDLINE (Via Pubmed): 1,475**

((Arthritis, Rheumatoid)) AND ((Anti Inflammatory Agents, Non Steroidal OR Analgesics, Anti-Inflammatory OR Glucocorticoid OR corticoids OR corticosteroids)) AND ((randomized controlled trial) OR randomization OR (control group) AND limit to human.

**EMBASE (Via Ovid): 5,956**

((Arthritis, Rheumatoid)) AND ((Anti Inflammatory Agents, Non Steroidal OR Analgesics, Anti-Inflammatory OR Glucocorticoid OR corticoids OR corticosteroids)) AND ((randomized controlled trial) OR randomization OR (control group) AND limit to human.

**CINAHAL (Via EBSCOhost): 123**

((Arthritis, Rheumatoid)) AND ((Anti Inflammatory Agents, Non Steroidal OR Analgesics, Anti-Inflammatory OR Glucocorticoid OR corticoids OR corticosteroids)) AND ((randomized controlled trial) OR randomization OR (control group)

**Web of science: 685**

((Arthritis, Rheumatoid)) AND ((Anti Inflammatory Agents, Non Steroidal OR Analgesics, Anti-Inflammatory OR Glucocorticoid OR corticoids OR corticosteroids)) AND ((randomized controlled trial) OR randomization OR (control group)

**Clinical trial.gov: 39**

(Arthritis, Rheumatoid) AND (Anti Inflammatory Agents, Non Steroidal OR Glucocorticoid)

**VHL: VIRTUAL HEALTH LIBRARY: 412**

(Arthritis, Rheumatoid) AND (Anti Inflammatory Agents, Non Steroidal OR Analgesics, Anti-Inflammatory OR Glucocorticoid OR corticoids OR corticosteroids) AND (randomized controlled trial OR randomization OR control group)

**TRIAL REGISTRY CLINICALTRIALS.GOV AND WORLD HEALTH ORGANIZATION INTERNATIONAL CLINICAL TRIALS REGISTRY PLATFORM: 17**

(Arthritis, Rheumatoid) AND (Anti Inflammatory Agents, Non Steroidal OR Glucocorticoid) AND (randomized controlled trial)
