# Supplementary material for: Use of corticoids and non-steroidal anti-inflammatories in the treatment of rheumatoid arthritis: Systematic review and network meta-analysis
Source: PLoS One. 2021 Apr 7;16(4):e0248866. doi: 10.1371/journal.pone.0248866 (PMC8026036; doi:10.1371/journal.pone.0248866)
Supplement: S2 File — (DOCX) [file pone.0248866.s003.docx]

S2 File. Main outcomes found in the articles not included in the meta-analysis

| Study | Sample size (N) | Interventions mg/day (N) | Pain | Physical function | N of painful  joints | N of swollen joints | Morning stiffness | Grip strength | Quality of life | Patients’ global assessment | | Physicians’ global assessment |
| --- | --- | --- | --- | --- | --- | --- | --- | --- | --- | --- | --- | --- |
| Bernhard et al., 1987^43^ | 234 | nabumetone 1,000, aspirin 900 | - | - | - | - | improvement in relation to baseline levels in both groups | improvement in relation to baseline levels in both groups | - | improvement in relation to baseline levels in both groups | | - |
| Collantes et al., 2002^45^ | 687 | placebo, etoricoxib 90, naproxen 1,000 | naproxen 1,000 was superior to placebo | etoricoxib 90 and naproxen 1,000 were more effective than placebo | - | - | - | - | - | - | | - |
| Emery et al., 1992^46^ | 284 | nabumetone 2,000, naproxen 1,000 | - | - | - | - | improvement in relation to baseline levels in both groups | - | - | - | | - |
| Emery et al., 1999^47^ | 497 | celecoxib 400, diclofenac 150 | - |  |  |  | improvement in relation to baseline levels in both groups |  |  |  | |  |
| Geusens et al., 2002^49^ | 1023 | placebo, naproxen 1,000 | - | naproxen 1,000 was superior to placebo | - | - | naproxen 1,000 was superior to placebo | - | - | - | | Naproxen 1,000 was superior to placebo |
| Geusens et al., 2004^50^ | 726 | naproxen 500, placebo | - |  |  |  |  |  |  |  | |  |
| Gibofsky et al., 2007^51^ | 340 | naproxen 1,000, placebo | - | - | - | - | naproxen 1,000 superior to placebo |  | Naproxen 1,000 was superior to placebo | | - | - |
| Jacob et al., 1986^52^ | 264 | placebo, etodolac 50, 100, 200, aspirin 3,900 | etodolac 100 and 200 were superior to placebo and etodolac 50 | etodolac 200 and aspirin 3,900 were superior to placebo | etodolac 200 and aspirin 3,900 were superior to placebo | etodolac 200 and aspirin 3,900 were superior to placebo | etodolac 200 and aspirin 3.900 were superior to placebo and etodolac 50 | etodolac 200 and aspirin 3,900 were not superior to placebo | - | etodolac 200 or aspirin 3,900 were superior to etodolac 50 | | etodolac 200 was superiot to placebo and aspirin 3,900 was superior to etodolac 50 |
| Kawai et al., 2010^42^ | 652 | placebo, ketoprofen 20 | ketoprofen 20 was not superior to placebo | - | - | - | - | - | - | - | | - |
| Kornasoff et al., 1996^53^ | 219 | aceclofenac 200, indomethacin 100 | - | improvement in relation to baseline levels in both groups | improvement in relation to baseline levels in both groups | improvement in relation to baseline levels in both groups | improvement in relation to baseline levels in both groups | - | - | improvement in relation to baseline levels in both groups | | improvement in relation to baseline levels in both groups |
| Krug et al., 2000^54^ | 344 | nabumetone 2,000, naproxen 1,000 | - | - | improvement in relation to baseline levels in both groups | improvement in relation to baseline levels in both groups | - | - | - | improvement in relation to baseline levels in both groups | | improvement in relation to baseline levels in both groups |
| Lightfoot, 1997^55^ | 361 | etodolac 400, 600, piroxicam 20 | - | - | improvement in relation to baseline levels in all groups | improvement in relation to baseline levels in all groups | improvement in relation to baseline levels in all groups | improvement in relation to baseline levels in both groups | - | - | | - |
| Matsumoto et al., 2002^56^ | 448 | placebo, etoricoxib 90, naproxen 1,000 | etoricoxib 90 and naproxen 1,000 was superior to placebo; etoricoxib 90 was superior to naproxen 1,000 | etoricoxib 90 was superior to placebo and naproxen 1,000 | - | - | - | - | - | - | | - |
| Pasero et al., 1995^57^ | 327 | aceclofenac 200, diclofenac 150 | - | - | - | - | improvement in relation to baseline levels in both groups | improvement in relation to baseline levels in both groups | - | - | | - |
| Perez ruiz; Alonso ruiz; Ansoleaga, 1996^58^ | 237 | aceclofenac 200, tenoxicam 20 | - | - | - | - | improvement in relation to baseline levels in both groups | improvement in relation to baseline levels in both groups | - | - | | - |
| Vasey et al., 1987^44^ | 318 | nabumetone 1,000, naproxen 500 | - | - | - | - | improvement in relation to baseline levels in both groups | improvement in relation to baseline levels in both groups | - | improvement in relation to baseline levels in both groups | | improvement in relation to baseline levels in both groups |
| Williams et al., 2006^60^ | 1093 | placebo, naproxen 500 | - | - | - | - | - | - | - | naproxen (1,000 mg/day) was superior to placebo (p<0.001 | | naproxen (1,000 mg/day) was superior to placebo (p<0.001 |
| Wojtulewski et al., 1996^61^ | 306 | meloxicam 7.5, naproxen 750 | - | - | - | - | improvement in relation to baseline levels in all groups | showed no statistically significant changes for variables from baseline and final | - | improvement in relation to baseline levels in both groups | | improvement in relation to baseline levels in both groups |
| Zhao et al., 2000^62^ | 688 | placebo, celecoxib 100, 200, 400, naproxen 1,000 | - | Celecoxib 200 and 400 and naproxen 1,000 were better than placebo; celecoxib 200 was superior to naproxen 1,000. | - | - | - | - | - | - | | - |
|  |  |  |  |  |  |  |  |  |  |  | |  |

**Notes.** Outcomes reported: 1: pain; 2: functional disability score; 3: swollen joint count; 4. tender joint count; 5: morning stiffness; 6: grip strength; 7: physician assessment; 8: patient assessment; 9: quality of life scale; 10: adverse events. NR: not reported.
