# Supplementary material for: Use of corticoids and non-steroidal anti-inflammatories in the treatment of rheumatoid arthritis: Systematic review and network meta-analysis
Source: PLoS One. 2021 Apr 7;16(4):e0248866. doi: 10.1371/journal.pone.0248866 (PMC8026036; doi:10.1371/journal.pone.0248866)
Supplement: S3 File — (DOCX) [file pone.0248866.s004.docx]

S3 File. GRADE for effectiveness and safety outcomes

CHART 1: Quality of evidence for pain outcome according to NMA GRADE

| **Comparison** | **Direct evidence** | | | **Indirect evidence** | | | **Network meta-analysis** | | |
| --- | --- | --- | --- | --- | --- | --- | --- | --- | --- |
|  | **SMD** | **95% confidence interval** | **Quality of evidence** | **SMD** | **95% confidence interval** | **Quality of evidence** | **SMD** | **95% confidence interval** | **Quality of evidence** |
| Placebo *v* tenoxicam 20 mg | - | - | - | -15.9 | (-41.5; 9.7) | Very Low**^,‡,¶^ | -15.9 | (-41.5; 9.7) | Very Low**^,‡,¶^ |
| Placebo *v* naproxen 750 mg | - | - | - | -11.0 | (-20.4; -0.2) | Very Low**^,‡,¶^ | -11.0 | (-20.4; -0.2) | Very Low**^,‡,¶^ |
| Placebo *v* naproxen 1,000 mg | 10.3 | (5.8; 14.8) | Very Low**^,†^ | -2.0 | (-58.7; 54.8) | Very Low**^,‡,†,¶^ | -10.3 | (-20.4; -0.2) | Very Low**^,†^ |
| Placebo *v* nabumetone 2,000 mg | - | - | - | -14.2 | (-32.0; 3.6) | Very Low**^,‡,¶^ | -14.2 | (-32.0; 3.6) | Very Low**^,‡,¶^ |
| Placebo *v* meloxicam 7,5 mg | 6.8 | (7.3; 12.1) | Moderate^†^ | -9.9 | (-63.0; 43.2) | Very Low^‡,†,¶^ | -6.6 | (-20.6; 7.2) | Moderate^†^ |
| Placebo *v* meloxicam 22,5 mg | 9.9 | (7.3; 15.2) | Moderate^†^ | -14.7 | (-75.6; 46.2) | Very Low^‡,†,¶^ | -9.8 | (-23.7; 4.1) | Moderate^†^ |
| Placebo *v* meloxicam 15 mg | 10.7 | (7.3; 16.0) | Moderate^†^ | -13.9 | (-74.8; 47.0) | Very Low^‡,†,¶^ | -10.6 | (-24.5; 3.3) | Moderate^†^ |
| Placebo *v* diclofenac 150 mg | 11.0 | (7.3; 16.3) | Moderate^†^ | -2.7 | (-48.1; 42.6) | Very Low^‡,†,¶^ | -10.8 | (-24.6; 3.0) | Moderate^†^ |
| Placebo *v* celecoxib 400 mg | - | - | - | -8.7 | (-28.7; 11.1) | Very Low*^,‡,¶^ | -8.7 | (-28.7; 11.1) | Very Low*^,‡,¶^ |
| Placebo *v* aceclofenac 200 mg | - | - | - | -13.9 | (-35.1; 7.2) | Very Low**^,‡,¶^ | -13.9 | (-35.1; 7.2) | Very Low**^,‡,¶^ |
| Tenoxicam 20 mg *v* naproxen 750 mg | - | - | - | 4.9 | (-24.9; 34.8) | Very Low**^,‡,¶^ | 4.9 | (-24.9; 34.8) | Very Low**^,‡,¶^ |
| Tenoxicam 20 mg *v* naproxen 1,000 mg | - | - | - | 5.6 | (-21.9; 33.1) | Very Low**^,‡,¶^ | 5.6 | (-21.9; 33.1) | Very Low**^,‡,¶^ |
| Tenoxicam 20 mg *v* nabumetone 2,000 mg | - | - | - | 1.7 | (-29.5; 32.9) | Very Low**^,‡,¶^ | 1.7 | (-29.5; 32.9) | Very Low**^,‡,¶^**^,‡,¶^ |
| Tenoxicam 20 mg *v* meloxicam 7,5 mg | - | - | - | 9.2 | (-16.7; 35.2) | Very Low**^,‡,¶^ | 9.2 | (-16.7; 35.2) | Very Low**^,‡,¶^ |
| Tenoxicam 20 mg *v* meloxicam 22,5 mg | - | - | - | 6.1 | (-19.8; 32.1) | Very Low**^,‡,¶^ | 6.1 | (-19.8; 32.1) | Very Low**^,‡,¶^ |
| Tenoxicam 20 mg *v* meloxicam 15 mg | - | - | - | 5.3 | (-20.7; 31.3) | Very Low**^,‡,¶^ | 5.3 | (-20.7; 31.3) | Very Low**^,‡,¶^ |
| Tenoxicam 20 mg *v* diclofenac 150 mg | - | - | - | 5.1 | (-16.5; 26.7) | Very Low**^,‡,¶^ | 5.1 | (-16.5; 26.7) | Very Low**^,‡,¶^ |
| Tenoxicam 20 mg *v* celecoxib 400 mg | - | - | - | 7.1 | (-18.8; 33.1) | Very Low**^,‡,¶^ | 7.1 | (-18.8; 33.1) | Very Low**^,‡,¶^ |
| Tenoxicam 20 mg *v* aceclofenac 200 mg | -2.0 | (-7.4; 3.4) | Very Low**^,‡,†^ | 28.6 | (-47.7;104) | Very Low**^,‡,†,¶^ | 1.9 | (-12.4; 16.4) | Very Low**^,‡,†,¶^ |
| Naproxen 750 mg *v* naproxen 1,000 mg | - | - | - | 0.7 | (-21.9; 23.3) | Very Low**^,‡,¶^ | 0.7 | (-21.9; 23.3) | Very Low**^,‡,¶^ |
| Naproxen 750 mg *v* nabumetone 2,000 mg | - | - | - | -3.2 | (-30.2; 23.7) | Very Low**^,‡,¶^ | -3.2 | (-30.2; 23.7) | Very Low**^,‡,¶^ |
| Naproxen 750 mg *v* meloxicam 7,5 mg | -4.3 | (-9.7; 1.1) | Very Low**^,‡,†^ | 13.4 | (-73.4; 100) | Very Low**^,‡,†,¶^ | 4.2 | (-10.4; 19.0) | Very Low**^,‡,†^ |
| Naproxen 750 mg *v* meloxicam 22,5 mg | - | - | - | 1.2 | (-19.5; 21.8) | Very Low**^,‡,¶^ | 1.2 | (-19.5; 21.8) | Very Low**^,‡,¶^ |
| Naproxen 750 mg *v* meloxicam 15 mg | - | - | - | 0.4 | (-10.3; 21.0) | Very Low**^,‡,¶^ | 0.4 | (-10.3; 21.0) | Very Low**^,‡,¶^ |
| Naproxen 750 mg *v* diclofenac 150 mg | - | - | - | 0.2 | (-20.5; 20.9) | Very Low**^,‡,¶^ | 0.2 | (-20.5; 20.9) | Very Low**^,‡,¶^ |
| Naproxen 750 mg *v* celecoxib 400 mg | - | - | - | 2.2 | (-22.9; 27.3) | Very Low**^,‡,¶^ | 2.2 | (-22.9; 27.3) | Very Low**^,‡,¶^ |
| Naproxen 750 mg *v* aceclofenac 200 mg | - | - | - | -2.9 | (-29.1; 23.2) | Very Low**^,‡,¶^ | -2.9 | (-29.1; 23.2) | Very Low**^,‡,¶^ |
| Naproxen 1,000 mg *v* nabumetone 2,000 mg | 4.0 | (-1.4; 9.4) | Very Low**^,‡,†^ | -20.5 | (-100; 59.7) | Very Low**^,‡,†,¶^ | -3.9 | (-18.6; 10.7) | Very Low**^,‡,†^ |
| Naproxen 1,000 mg *v* meloxicam 7,5 mg | - | - | - | 3.6 | (-13.6; 20.8) | Very Low**^,‡,¶^ | 3.6 | (-13.6; 20.8) | Very Low**^,‡,¶^ |
| Naproxen 1,000 mg *v* meloxicam 22,5 mg | - | - | - | 0.5 | (-16.7; 17.7) | Very Low**^,‡,¶^ | 0.5 | (-16.7; 17.7) | Very Low**^,‡,¶^ |
| Naproxen 1,000 mg *v* meloxicam 15 mg | - | - | - | -0.3 | (-17.5; 16.7) | Very Low**^,‡,¶^ | -0.3 | (-17.5; 16.7) | Very Low**^,‡,¶^ |
| Naproxen 1,000 mg *v* diclofenac 150 mg | - | - | - | -0.5 | (-17.7; 16.6) | Very Low**^,‡,¶^ | -0.5 | (-17.7; 16.6) | Very Low**^,‡,¶^ |
| Naproxen 1,000 mg *v* celecoxib 400 mg | - | - | - | 1.5 | (-10.8; 23.8) | Very Low**^,‡,¶^ | 1.5 | (-10.8; 23.8) | Very Low**^,‡,¶^ |
| Naproxen 1,000 mg *v* aceclofenac 200 mg | - | - | - | -3.6 | (-27.1; 19.8) | Very Low**^,‡,¶^ | -3.6 | (-27.1; 19.8) | Very Low**^,‡,¶^ |
| Nabumetone 2,000 mg *v* meloxicam 7,5 mg | - | - | - | 7.5 | (-15.0; 30.1) | Very Low**^,‡,¶^ | 7.5 | (-15.0; 30.1) | Very Low**^,‡,¶^ |
| Nabumetone 2,000 mg *v* meloxicam 22,5 mg | - | - | - | 4.4 | (-18.2; 27.0) | Very Low**^,‡,¶^ | 4.4 | (-18.2; 27.0) | Very Low**^,‡,¶^ |
| Nabumetone 2,000 mg *v* meloxicam 15 mg | - | - | - | 3.6 | (-19.0; 26.2) | Very Low**^,‡,¶^ | 3.6 | (-19.0; 26.2) | Very Low**^,‡,¶^ |
| Nabumetone 2,000 mg *v* diclofenac 150 mg | - | - | - | 3.4 | (-19.1; 26.0) | Very Low**^,‡,¶^ | 3.4 | (-19.1; 26.0) | Very Low**^,‡,¶^ |
| Nabumetone 2,000 mg *v* celecoxib 400 mg | - | - | - | 5.4 | (-21.3; 32.2) | Very Low**^,‡,¶^ | 5.4 | (-21.3; 32.2) | Very Low**^,‡,¶^ |
| Nabumetone 2,000 mg *v* aceclofenac 200mg | - | - | - | 0.3 | (-27.4; 28.0) | Very Low**^,‡,¶^ | 0.3 | (-27.4; 28.0) | Very Low**^,‡,¶^ |
| Meloxicam 7,5 mg *v* meloxicam 22,5 mg | 3.1 | (2.3; 8.5) | High | 20.8 | (-66; 107.5) | Low^‡,¶^ | -3.1 | (-17.6; 11.4) | High |
| Meloxicam 7,5 mg *v* meloxicam 15 mg | 3.9 | (-1.5; 9.3) | Moderate^‡^ | 21.5 | (-65.1; 108) | Low^‡,¶^ | -3.9 | (-18.4; 10.6) | Moderate^‡^ |
| Meloxicam 7,5 mg *v* diclofenac 150 mg | 4.2 | (-1.2; 9.6) | Low^‡,†^ | -11.8 | (-72.7; 49.2) | Very Low^‡,†,¶^ | -4.1 | (-18.6; 10.4) | Low^‡,†^ |
| Meloxicam 7,5 mg *v* celecoxib 400 mg | - | - | - | -2.1 | (-22.5; 18.3) | Very Low*^,‡,¶^ | -2.1 | (-22.5; 18.3) | Very Low*^,‡,¶^ |
| Meloxicam 7,5 mg *v* aceclofenac 200 mg | - | - | - | -7.2 | (-28.9; 14.4) | Very Low**^,‡,¶^ | -7.2 | (-28.9; 14.4) | Very Low**^,‡,¶^ |
| Meloxicam 22,5 mg *v* meloxicam 15 mg | - | - | - | -0.8 | (-15.3; 13.7) | Low^‡,¶^ | -0.8 | (-15.3; 13.7) | Low^‡,¶^ |
| Meloxicam 22,5 mg *v* diclofenac 150 mg | 0.3 | (-5.1; 5.7) | Moderate^‡^ | 26.5 | (-38.9; 91.9) | Low^‡,¶^ | -1.0 | (-15.5; 13.5) | Moderate^‡^ |
| Meloxicam 22,5 mg *v* celecoxib 400 mg | - | - | - | 1.0 | (-19.3; 21.4) | Very Low*^,‡,¶^ | 1.0 | (-19.3; 21.4) | Very Low*^,‡,¶^ |
| Meloxicam 22,5 mg *v* aceclofenac 200 mg | - | - | - | -4.1 | (-25.8; 17.5) | Very Low**^,‡,¶^ | -4.1 | (-25.8; 17.5) | Very Low**^,‡,¶^ |
| Meloxicam 15 mg *v* diclofenac 150 mg | 1.1 | (-4.3; 6,5) | Low^‡,†^ | -25.7 | (-91.1; 39.6) | Very Low^‡,†,¶^ | -0.2 | (-14.7; 14.3) | Low^‡,†^ |
| Meloxicam 15 mg *v* celecoxib 400 mg | - | - | - | 1.8 | (-18.5; 22.2) | Very Low*^,‡,¶^ | 1.8 | (-18.5; 22.2) | Very Low*^,‡,¶^ |
| Meloxicam 15 mg *v* aceclofenac 200 mg | - | - | - | -3.3 | (-25.0; 18.3) | Very Low**^,‡,¶^ | -3.3 | (-25.0; 18.3) | Very Low**^,‡,¶^ |
| Diclofenac 150 mg *v* celecoxib 400 mg | -2.0 | (-7.4; 3.4) | Low*^,‡^ | -22.0 | (-114; 70.5) | Very Low*^,‡,¶^ | 2.0 | (-12.3; 16.4) | Low*^,‡^ |
| Diclofenac 150 mg *v* aceclofenac 200 mg | 3.3 | (-2.4; 9.0) | Very Low**^,‡,†^ | -12.9 | (65.6; 39.8) | Very Low**^,†,¶^ | -3.1 | (-19.3; 13.0) | Very Low**^,‡,†^ |
| Celecoxib 400 mg *v* aceclofenac 200 mg | - | - | - | -5.1 | (-26.8; 16.4) | Very Low**^,‡,¶^ | -5.1 | (-26.8; 16.4) | Very Low**^,‡,¶^ |

*risk of bias moderate. ** risk of bias High. ‡Imprecision. †Inconsistency. ¶ Indirectness because of questionable comparability of trial populations.

CHART 2: Quality of evidence for physical function outcome, according to NMA GRADE

| **Comparison** | **Direct evidence** | | | **Indirect evidence** | | | **Network meta-analysis** | | |
| --- | --- | --- | --- | --- | --- | --- | --- | --- | --- |
|  | **SMD** | **95% confidence interval** | **Quality of evidence** | **SMD** | **95% confidence interval** | **Quality of evidence** | **SMD** | **95% confidence interval** | **Quality of evidence** |
| Placebo *v* naproxen 1,000 mg | - | - | - | -0.1 | (-0.2; -0.0) | Very Low**^,¶^ | -0.1 | (-0.2; -0.0) | **Very Low****^,¶^ |
| Placebo *v* meloxicam 7.5 mg | - | - | - | -0.0 | (-0.2; 0.1) | Low^‡,¶^ | -0.0 | (-0.2; 0.1) | Low^‡,¶^ |
| Placebo *v* meloxicam 22.5 mg | 0.1 | (-0.4; 0.7) | Low^‡,†^ | -0.0 | (-15.1; 15.1) | Very Low ^‡,†,¶^ | -0.1 | (-0.3; 0.0) | Low |
| Placebo *v* meloxicam 15 mg | 0.1 | (-0.4; 0.6) | Low^‡,†^ | -0.0 | (-15.2; 15.1) | Very Low^‡,†,¶^ | -0.1 | (-0.3; 0.0) | Low |
| Placebo *v* diclofenac 150 mg | 0.1 | (-0.4; 0.6) | Low^‡,†^ | -0.0 | (-10.7; 10.7) | Very Low^‡,†,¶^ | -0.1 | (-0.2; 0.0) | Low |
| Placebo *v* celecoxib 400 mg | - | - | - | -0.1 | (-0.3; 0.1) | Very Low*^,‡,¶^ | -0.1 | (-0.3; 0.1) | Very Low*^,‡,¶^ |
| Naproxen 1,000 mg *v* meloxicam 7.5 mg | - | - | - | 0.1 | (-0.1; 0.2) | Very Low**^,‡,¶^ | 0.1 | (-0.1; 0.2) | Very Low**^,‡,¶^ |
| Naproxen 1,000 mg *v* meloxicam 22.5 mg - | | - | - | 0.0 | (-0.2; 0.2) | Very Low**^,‡,¶^ | 0.0 | (-0.2; 0.2) | Very Low**^,‡,¶^ |
| Naproxen 1,000 mg *v* meloxicam 15 mg | - | - | - | 0.0 | (-0.1; 0.2) | Very Low**^,‡,¶^ | 0.0 | (-0.1; 0.2) | Very Low**^,‡,¶^ |
| Naproxen 1,000 mg *v* diclofenac 150 mg | - | - | - | 0.0 | (-0.1; 0.2) | Very Low**^,‡,¶^ | 0.0 | (-0.1; 0.2) | Very Low**^,‡,¶^ |
| Naproxen 1,000 mg *v* celecoxib 400 mg | - | - | - | 0.0 | (-0.2; 0.3) | Very Low**^,‡,¶^ | 0.0 | (-0.2; 0.3) | Very Low**^,‡,¶^ |
| Meloxicam 7.5 mg *v* meloxicam 22.5 mg | - | - | - | -0.1 | (-0.2; 0.1) | Low^‡,¶^ | -0.1 | (-0.2; 0.1) | Low^‡,¶^ |
| Meloxicam 7.5 mg *v* meloxicam 15 mg | - | - | - | -0.0 | (-0.2; 0.1) | Low^‡,¶^ | -0.0 | (-0.2; 0.1) | Low^‡,¶^ |
| Meloxicam 7.5 mg *v* diclofenac 150 mg | 0.0 | (-0.5; 0.5) | Low^‡,†^ | -0.1 | (-15.3; 15.0) | Very Low^‡,†,¶^ | -0.0 | (-0.1; 0.1) | Low |
| Meloxicam 7.5 mg *v* celecoxib 400 mg | - | - | - | -0.0 | (-0.2; 0.2) | Very Low*^,‡,¶^ | -0.0 | (-0.2; 0.2) | Very Low*^,‡,¶^ |
| Meloxicam 22.5 mg *v* meloxicam 15 mg | - | - | - | 0.0 | (-0.1; 0.1) | Low*^,‡,¶^ | 0.0 | (-0.1; 0.1) | Low*^,‡,¶^ |
| Meloxicam 22.5 mg *v* diclofenac 150 mg | -0.0 | (-0.6; 0.4) | Moderate^‡^ | -0.2 | (-15.4; 15.0) | Very Low^‡,†,¶^ | 0.0 | (-0.0; 0.2) | Moderate |
| Meloxicam 22.5 mg *v* celecoxib 400 mg | - | - | - | 0.0 | (-0.2; 0.3) | Very Low*^,‡,¶^ | 0.0 | (-0.2; 0.3) | Very Low*^,‡,¶^ |
| Diclofenac 150 mg *v* celecoxib 400 mg | 0.1 | (-0.5; 0.6) | Low*^,‡^ | 0.1 | (-15.0; 15.3) | Very Low*^,‡,¶^ | 0.0 | (-0.2; 0.1) | Low |
| Meloxicam 15 mg *v* diclofenac 150 mg | -0.0 | (-0.6; 0.5) | Moderate^‡^ | -0.2 | (-15.4; 15.0) | Low^‡,¶^ | 0.0 | (-0.1; 0.2) | Moderate |
| Meloxicam 15 mg *v* celecoxib 400 mg | - | - | - | 0.0 | (-0.2; 0.3) | Very Low*^,‡,¶^ | 0.0 | (-0.2; 0.3) | Very Low*^,‡,¶^ |

*risk of bias moderate. ** risk of bias High. ‡Imprecision. †Inconsistency. ¶ Indirectness because of questionable comparability of trial populations

CHART 3: Quality of evidence for number of tender/painful joints outcome, according to NMA GRADE

| **Comparison** | **Direct evidence** | | | **Indirect evidence** | | | **Network meta-analysis** | | |
| --- | --- | --- | --- | --- | --- | --- | --- | --- | --- |
|  | **SMD** | **95% confidence interval** | **Quality of evidence** | **SMD** | **95% confidence interval** | **Quality of evidence** | **SMD** | **95% confidence interval** | **Quality of evidence** |
| Placebo *v* naproxen 750 mg | - | - | - | -5.3 | (-12.2; 1.5) | Very Low**^,‡,¶^ | -5.3 | (-12.2; 1.5) | Very Low**^,‡,¶^ |
| Placebo *v* naproxen 1,000 mg | - | - | - | -3.5 | (-5.1; -1.9) | Very Low**^,¶^ | -3.5 | (-5.1; -1.9) | **Very Low****^,¶^ |
| Placebo *v* meloxicam 7.5 mg | 1.6 | (-1.0; 4.2) | Low^‡,†^ | -0.7 | (-19.3; 17.7) | Very Low^‡,,†,¶^ | -1.6 | (-5.1; 1.9) | Low |
| Placebo *v* meloxicam 22.5 mg | 1.6 | (-1.0; 4.2) | Low^‡,†^ | -3.0 | (-26.1; 20.5) | Very Low^‡,,†,¶^ | -1.6 | (-5.1; 1,9) | Low |
| Placebo *v* meloxicam 15 mg | 0.9 | (-1.7; 3.5) | Low^‡,†^ | -3.0 | (-26.8; 19.8) | Very Low^‡,,†,¶^ | -0.9 | (-4.4; 2.6) | Low |
| Placebo *v* etoricoxib 90 mg | 5.0 | (2.5; 6.9) | Very Low**^,‡^ | 7.0 | (3.5; 10.5) | Very Low**^,¶^ | -4.9 | (-7.1; -2.8) | **Very Low** |
| Placebo *v* diclofenac 150 mg | 2.3 | (-0.3; 4.9) | - | -1.0 | (-6.3; 5.1) | Very Low^‡,†,¶^ | -2.3 | (-5.8; 1.2) | Very Low^‡,†,¶^ |
| Placebo *v* celecoxib 400 mg | - | - | - | -2.8 | (-8.4; 2.8) | Very Low*^,‡,¶^ | -2.8 | (-8.4; 2.8) | Very Low*^,‡,¶^ |
| Naproxen 750 mg *v* naproxen 1,000 mg | - | - | - | 1.8 | (-5.2; 8.8) | Very Low**^,‡,¶^ | 1.8 | (-5.2; 8.8) | Very Low**^,‡,¶^ |
| Naproxen 750 mg *v* meloxicam 7.5 mg | -3.7 | (-7.1; -0.3) | Low** | -0.5 | (-28.3; 27.2) | Very Low**^,‡,¶^ | 3.7 | (-2.1; 9.6) | Low** |
| Naproxen 750 mg *v* meloxicam 15 mg | - | - | - | 4.4 | (-2.4; 11.3) | Very Low**^,‡,¶^ | 4.4 | (-2.4; 11.3) | Very Low**^,‡,¶^ |
| Naproxen 750 mg *v* meloxicam 22.5 mg | - | - | - | 3.7 | (-3.1; 10.6) | Very Low**^,‡,¶^ | 3.7 | (-3.1; 10.6) | Very Low**^,‡,¶^ |
| Naproxen 750 mg *v* etoricoxib 90 mg | - | - | - | 0.3 | (-6.8; 7.5) | Very Low**^,‡,¶^ | 0.3 | (-6.8; 7.5) | Very Low**^,‡,¶^ |
| Naproxen 750 mg *v* diclofenac 150 mg | - | - | - | 3.0 | (-3.8; 9.9) | Very Low**^,‡,¶^ | 3.0 | (-3.8; 9.9) | Very Low**^,‡,¶^ |
| Naproxen 750 mg *v* celecoxib 400 mg | - | - | - | 2.5 | (-5.6; 10.7) | Very Low**^,‡,¶^ | 2.5 | (-5.6; 10.7) | Very Low**^,‡,¶^ |
| Naproxen 1,000 mg *v* meloxicam 7.5 mg | - | - | - | 1.9 | (-1.9; 5.8) | Very Low**^,‡,¶^ | 1.9 | (-1.9; 5.8) | Very Low**^,‡,¶^ |
| Naproxen 1,000 mg *v* meloxicam 22.5 mg | - | - | - | 1.9 | (-1.9; 5.8) | Very Low**^,‡,¶^ | 1.9 | (-1.9; 5.8) | Very Low**^,‡,¶^ |
| Naproxen 1,000 mg *v* meloxicam 15 mg | - | - | - | 2.6 | (-1.2; 6.5) | Very Low**^,‡,¶^ | 2.6 | (-1.2; 6.5) | Very Low**^,‡,¶^ |
| Naproxen 1,000 mg *v* etoricoxib 90 mg | 2.0 | (-0.5; 4.0) | Very Low**^,‡,†^ | -0.5 | (-4.1; 3.0) | Very Low**^,‡,†,¶^ | -1.4 | (-3.6; 0.7) | Very Low**^,‡,†^ |
| Naproxen 1,000 mg *v* diclofenac 150 mg | - | - | - | 1.2 | (-2.6; 5.1) | Very Low**^,‡,¶^ | 1.2 | (-2.6; 5.1) | Very Low**^,‡,¶^ |
| Naproxen 1,000 mg *v* celecoxib 400 mg | - | - | - | 0.7 | (-5.1; 6.6) | Very Low**^,‡,¶^ | 0.7 | (-5.1; 6.6) | Very Low**^,‡,¶^ |
| Meloxicam 7.5 mg *v* meloxicam 22.5 mg | 2.6 | (0.0; 5.2) | High | 0 | (-3.5; 3.5) | Low^‡,¶^ | 0 | (-3.5; 3.5) | High |
| Meloxicam 7.5 mg *v* meloxicam 15 mg | -0.7 | (-3.3; 1.9) | Low^‡,†^ | 2.0 | (-25.2; 30.2) | Very Low^‡,†,¶^ | 0.7 | (-2.8; 4.2) | Low^‡,†^ |
| Meloxicam 7.5 mg *v* etoricoxib 90 mg | - | - | - | 3.3 | (-7.5; 0.7) | Very Low**^,‡,¶^ | 3.3 | (-7.5; 0.7) | Very Low**^,‡,¶^ |
| Meloxicam 7.5 mg *v* diclofenac 150 mg | 0.7 | (-1.9; 3.3) | Low^‡,†^ | -0.4 | (-23.8; 22.8) | Very Low^‡,†,¶^ | -0.7 | (-4.2; 2.8) | Low^‡,†^ |
| Meloxicam 7.5 mg *v* celecoxib 400 mg | - | - | - | -1.2 | (-6.8; 4.4) | Very Low*^,‡,¶^ | -1.2 | (-6.8; 4.4) | Very Low*^,‡,¶^ |
| Meloxicam 22.5 mg *v* meloxicam 15 mg | - | - | - | 0.7 | (-2.8; 4.2) | Low^‡,¶^ | 0.7 | (-2.8; 4.2) | Low^‡,¶^ |
| Meloxicam 22.5 mg *v* etoricoxib 90 mg | - | - | - | -3.3 | (-7.5; 0.7) | Very Low**^,‡,¶^ | -3.3 | (-7.5; 0.7) | Very Low**^,‡,¶^ |
| Meloxicam 22.5 mg *v* diclofenac 150 mg | 0.7 | (-1.9; 3.3) | Low^‡,†^ | -5.0 | (-32.6; 22.8) | Very Low^‡,†,¶^ | -0.7 | (-4.2; 2.8) | Low^‡,†^ |
| Meloxicam 22.5 mg *v* celecoxib 400 mg | - | - | - | -1.2 | (-6.8; 4.4) | Very Low*^,‡,¶^ | -1.2 | (-6.8; 4.4) | Very Low*^,‡,¶^ |
| Meloxicam 15 mg *v* etoricoxib 90 mg | - | - | - | -4.0 | (-8.2; 0.0) | Very Low**^,‡,¶^ | -4.0 | (-8.2; 0.0) | Very Low**^,‡,¶^ |
| Meloxicam 15 mg *v* diclofenac 150 mg | 1.4 | (-1.2; 4.0) | Low^‡,†^ | -4.0 | (-31.9; 23.5) | Very Low^‡,†,¶^ | -1.4 | (-4.9; 2.1) | Low^‡,†^ |
| Meloxicam 15 mg *v* celecoxib 400 mg | - | - | - | -1.9 | (-7.5; 3.7) | Very Low*^,‡,¶^ | -1.9 | (-7.5; 3.7) | Very Low*^,‡,¶^ |
| Etoricoxib 90 mg *v* diclofenac 150 mg | - | - | - | 2.7 | (-1.4; 6.8) | Very Low**^,‡,¶^ | 2.7 | (-1.4; 6.8) | Very Low**^,‡,¶^ |
| Etoricoxib 90 mg *v* celecoxib 400 mg | - | - | - | 2.1 | (-3.9; 8.2) | Very Low**^,‡,¶^ | 2.1 | (-3.9; 8.2) | Very Low**^,‡,¶^ |
| Diclofenac 150 mg *v* celecoxib 400mg | 0.5 | (-2.4; 3.4) | Very Low*^,‡,†^ | -5.0 | (-32.8; 22.6) | Very Low*^,‡,†,¶^ | -0.5 | (-4.9; 3.9) | Very Low*^,‡,†^ |

*risk of bias moderate. ** risk of bias High. ‡Imprecision. †Inconsistency. ¶ Indirectness because of questionable comparability of trial populations

CHART 4: Quality of evidence for number of swollen joints outcome, according to NMA GRADE

| **Comparison** | **Direct evidence** | | | **Indirect evidence** | | | **Network meta-analysis** | | |
| --- | --- | --- | --- | --- | --- | --- | --- | --- | --- |
|  | **SMD** | **95% confidence interval** | **Quality of evidence** | **SMD** | **95% confidence interval** | **Quality of evidence** | **SMD** | **95% confidence interval** | **Quality of evidence** |
| Placebo *v* naproxen 750 mg |  |  |  | -6.7 | (-11.0; -2.4) | Very Low**^,¶^ | -6.7 | (-11.0; -2.4) | Very Low** |
| Placebo *v* naproxen 1,000 mg |  |  |  | -1.5 | (-2.3; -0.7) | Very Low**^,¶^ | -1.5 | (-2.3; -0.7) | Very Low** |
| Placebo *v* meloxicam 7.5 mg | 1.5 | (-0.4; 3.4) | Low^‡,†^ | -0.1 | (-18.6; 18.4) | Very Low^‡,†,¶^ | -1.5 | (-3.3; 0.3) | Low^‡,†^ |
| Placebo *v* meloxicam 22.5 mg | 1.4 | (-0.5; 3.3) | Low^‡,†^ | -1.1 | (-24.4; 22.2) | Very Low^‡,†,¶^ | -1.4 | (-3.2; 0.4) | Low^‡,†^ |
| Placebo *v* meloxicam 15 mg | 0.2 | (-1.7; 2.1) | Low^‡,†^ | -2.3 | (-25.6; 21.0) | Low^†,¶^ | -0.2 | (-2.0; 1.64) | Low^‡,†^ |
| Placebo *v* etoricoxib 90 mg | 2.5 | (1.1; 3.9) | Low** | 3.5 | (1.0; 6.0) | Very Low**^,¶^ | -2.6 | (-3.2; -1.7) | Low** |
| Placebo *v* diclofenac 150 mg | 0.7 | (-1.2; 2.6) | Low^‡,†^ | -0.7 | (-19.2; 17.8) | Very Low^‡,†,¶^ | -0.7 | (-2.5; 1.4) | Low^‡,†^ |
| Placebo *v* celecoxib 400 mg |  |  |  | -1 | (-4.1; 2.1) | Low*^,¶^ | -1 | (-4.1; 2.1) | Low* |
| Naproxen 750 mg *v* naproxen 1.000 mg |  |  |  | 5.2 | (0.8; 9.6) | Very Low**^,¶^ | 5.2 | (0.8; 9.6) | Very Low** |
| Naproxen 750 mg *v* meloxicam 7.5 mg | -5.2 | (-8.0; -2.4) | Low** | -2.2 | (-29.9; 25.5) | Very Low**^‡,¶^ | 5.2 | (1.4; 9.1) | Low** |
| Naproxen 750 mg *v* meloxicam 22.5 mg |  |  |  | 5.3 | (1.0; 9.3) | Very Low**^,¶^ | 5.3 | (1.0; 9.3) | Very Low** |
| Naproxen 750 mg *v* meloxicam 15 mg |  |  |  | 6.4 | (2.2; 10.8) | Very Low**^,¶^ | 6.4 | (2.2; 10.8) | Very Low** |
| Naproxen 750 mg *v* etoricoxib 90 mg |  |  |  | 4.1 | (-0.3; 8.5) | Very Low**^,‡,¶^ | 4.1 | (-0.3; 8.5) | Very Low |
| Naproxen 750 mg *v* diclofenac 150 mg |  |  |  | 6 | (1.7; 10.3) | Very Low**^,¶^ | 6 | (1.7; 10.3) | Very Low** |
| Naproxen 750 mg *v* celecoxib 400 mg |  |  |  | 5.7 | (0.8; 10.7) | Very Low**^‡,¶^ | 5.7 | (0.8; 10.7) | Very Low**^‡^ |
| Naproxen 1,000 mg *v* meloxicam 7.5 mg |  |  |  | 0 | (-1.9; 2.0) | Very Low**^,¶^ | 0 | (-1.9; 2.0) | Very Low** |
| Naproxen 1,000 mg *v* meloxicam 22.5 mg |  |  |  | 0.1 | (-1.9; 2.1) | Very Low**^,¶^ | 0.1 | (-1.9; 2.1) | Very Low** |
| Naproxen 1,000 mg *v* meloxicam 15 mg |  |  |  | 1.3 | (0.7; 3.3) | Very Low**^,¶^ | 1.3 | (0.7; 3.3) | Very Low** |
| Naproxen 1,000 mg *v* etoricoxib 90 mg | 1.2 | (-0.2; 2.6) | Very Low**^,‡^ | 0.2 | (-2.3; 2.7) | Very Low**^,¶^ | -1.1 | (-2.0; -0.1) | Very Low** |
| Naproxen 1,000 mg *v* diclofenac 150 mg |  |  |  | 0.8 | (-1.9; 2.8) | Very Low**^,¶^ | 0.8 | (-1.9; 2.8) | Very Low** |
| Naproxen 1,000 mg *v* celecoxib 400 mg |  |  |  | 0.5 | (-2.7; 3.7) | Very Low**^,¶^ | 0.5 | (-2.7; 3.7) | Very Low** |
| Meloxicam 7.5 mg *v* meloxicam 22.5 mg | -0.1 | (-2.0; 1.8) | Low^‡,†^ | 2.9 | (-24.8; 30.6) | Low^†,¶^ | 0.1 | (-1.7; 1.9) | Low^†^ |
| Meloxicam 7.5 mg *v* meloxicam 15 mg | -1.3 | (-3.2; 0.6) | Low^‡,†^ | 1.7 | (-26.0; 29.4) | Low^†,¶^ | 1.3 | (-0.5; 3.1) | Low^†^ |
| Meloxicam 7.5 mg *v* etoricoxib 90 mg |  |  |  | -1.1 | (-3.1; 0.9) | Very Low**^,¶^ | -1.1 | (-3.1; 0.9) | Very Low** |
| Meloxicam 7.5 mg *v* diclofenac 150 mg | -0.8 | (-2.7; 1.1) | Moderate^‡^ | -0.3 | (-23.7; 23.1) | Moderate^,¶^ | 0.8 | (1.0; 2.6) | Moderate^‡^ |
| Meloxicam 7.5 mg *v* celecoxib 400 mg |  |  |  | 0.5 | (-2.6; 3.6) | Low*^,¶^ | 0.5 | (-2.6; 3.6) | Low* |
| Meloxicam 22.5 mg *v* meloxicam 15 mg |  |  |  | 1.2 | (-6.4; 3.0) | Moderate^,¶^ | 1.2 | (-6.4; 3.0) | Moderate |
| Meloxicam 22.5 mg *v* etoricoxib 90 mg |  |  |  | -1.2 | (-3.2; 0.8) | Very Low**^,¶^ | -1.2 | (-3.2; 0.8) | Very Low |
| Meloxicam 22.5 mg *v* diclofenac 150 mg | -0.7 | (-2.6; 1.2) | Moderate^‡^ | -2.7 | (-30.4; 25.0) | Moderate^,¶^ | 0.7 | (-1.1; 2.5) | Moderate^‡^ |
| Meloxicam 22.5 mg *v* celecoxib 400 mg |  |  |  | 0.4 | (-2.7; 3.5) | Low*^,¶^ | 0.4 | (-2.7; 3.5) | Low* |
| Meloxicam 15 mg *v* etoricoxib 90 mg |  |  |  | -2.4 | (-4.5; 0.4) | Very Low**^,¶^ | -2.4 | (-4.5; 0.4) | Very Low** |
| Meloxicam 15 mg *v* diclofenac 150 mg | 0.5 | (-1.4; 2.4) | Low^‡,†^ | -1.5 | (-29.2; 26.2) | Low^,¶^ | -0.5 | (-2.3; 1.3) | Low^‡,†^ |
| Meloxicam 15 mg *v* celecoxib 400 mg |  |  |  | -0.8 | (-3.9; 2.3) | Low*^,¶^ | -0.8 | (-3.9; 2.3) | Low* |
| Etoricoxib 90 mg *v* diclofenac 150 mg |  |  |  | 1.9 | (-0.1; 4.0) | Very Low**^,†^ | 1.9 | (-0.1; 4.0) | Very Low** |
| Etoricoxib 90 mg *v* celecoxib 400 mg |  |  |  | 1.6 | (-1.6; 4.9) | Very Low**^,¶^ | 1.6 | (-1.6; 4.9) | Very Low** |
| Diclofenac 150 mg *v* celecoxib 400mg | 0.3 | (-1.9; 2.5) | Very Low*^,‡,†^ | -1.7 | (-29.4; 26.0) | Very Low*^,†,¶^ | -0.3 | (-2.8; 2.2) | Very Low*^,‡,†^ |

*risk of bias moderate. ** risk of bias High. ‡Imprecision. †Inconsistency. ¶ Indirectness because of questionable comparability of trial populations

CHART 5: Quality of evidence for patients’ global assessment outcome, according to NMA GRADE

| **Comparison** | **Direct evidence** | | | **Indirect evidence** | | | **Network meta-analysis** | | |
| --- | --- | --- | --- | --- | --- | --- | --- | --- | --- |
|  | **SMD** | **95% confidence interval** | **Quality of evidence** | **SMD** | **95% confidence interval** | **Quality of evidence** | **SMD** | **95% confidence interval** | **Quality of evidence** |
| Placebo x naproxen 1,000 mg | - | - | - | -11.1 | (-15.7; -6.5) | Very Low**^,¶^ | -11.1 | (-15.7; -6.5) | Very Low**^,¶^ |
| Placebo x meloxicam 7.5 mg | 6.2 | (1.9; 10.5) | Moderate^†^ | -14.0 | (-41.7; 13.7) | Very Low^‡,†,¶^ | -6.2 | (-15.6; 3.2) | Moderate^†^ |
| Placebo x meloxicam 22.5 mg | 9.1 | (4.8; 13.3) | Moderate^†^ | -11.1 | (-38.8; 16.6) | Very Low^‡,†,¶^ | -9.1 | (-18.5; 0.3) | Moderate^†^ |
| Placebo x meloxicam 15 mg | 8.7 | (4.4; 12.9) | Moderate^†^ | -11.5 | (-39.2; 16.2) | Very Low^‡,†,¶^ | -8.7 | (-18.1; 0.7) | Moderate^†^ |
| Placebo x etoricoxib 90 mg | 14.4 | (10.6; 18.1) | Low** | 14.0 | (7.6; 19.9) | Very Low**^,¶^ | 14.3 | (-20.2; -8.4) | Low** |
| Placebo x diclofenac 150 mg | 10.1 | (5.8; 14.3) | High | 4.0 | (-15.6; 23.6) | Low^‡ ,¶^ | -10.0 | (-19.5; -0.6) | High |
| Placebo x celecoxib 400 mg | - | - | - | -10.0 | (-22.7; 2.6) | Very Low*^,‡,¶^ | -10.0 | (-22.7; 2.6) | Very Low*^,‡,¶^ |
| Naproxen 1,000 mg x meloxicam 7.5 mg | - | - | - | 4.9 | (-5.6; 15.4) | Very Low**^,‡,¶^ | 4.9 | (-5.6; 15.4) | Very Low**^,‡,¶^ |
| Naproxen 1,000 mg x meloxicam 22.5 mg | - | - | - | 2.0 | (-8.5; 12.5) | Very Low**^,‡,¶^ | 2.0 | (-8.5; 12.5) | Very Low**^,‡,¶^ |
| Naproxen 1,000 mg x meloxicam 15 mg | - | - | - | 2.4 | (-8.1; 12.9) | Very Low**^,‡,¶^ | 2.4 | (-8.1; 12.9) | Very Low**^,‡,¶^ |
| Naproxen 1,000 mg x etoricoxib 90 mg | 3.1 | (-0.6; 6.8) | Very Low**^,‡,†^ | 3.6 | (-2.6; 9.7) | Very Low**^,‡,¶^ | -3.2 | (-8.9; 2.5) | Very Low**^,‡,†^ |
| Naproxen 1,000 mg x diclofenac 150 mg | - | - | - | 1.0 | (-9.4; 11.5) | Very Low**^,‡,¶^ | 1.0 | (-9.4; 11.5) | Very Low**^,‡,¶^ |
| Naproxen 1,000 mg x celecoxib 400 mg | - | - | - | 1.0 | (-12.5; 14.5) | Very Low**^,‡,¶^ | 1.0 | (-12.5; 14.5) | Very Low**^,‡,¶^ |
| Meloxicam 7.5 mg x meloxicam 22.5 mg | - | - | - | -2.9 | (-12.3; 6.5) | Low^‡,¶^ | -2.9 | (-12.3; 6.5) | Low^‡,¶^ |
| Meloxicam 7.5 mg x meloxicam 15 mg | - | - | - | -2.5 | (-11.9; 6.9) | Low^‡,¶^ | -2.5 | (-11.9; 6.9) | Low^‡,¶^ |
| Meloxicam 7.5 mg x etoricoxib 90 mg | - | - | - | -8.1 | (-19.3; 3.0) | Very Low**^,‡,¶^ | -8.1 | (-19.3; 3.0) | Very Low**^,‡,¶^ |
| Meloxicam 7.5 mg x diclofenac 150 mg | 3.9 | (-0.4; 8.1) | Low^‡,†^ | -16.3 | (-44.0; 11.4) | Very Low^‡,†,¶^ | -3.9 | (-13.3; 5.5) | Low^‡,†^ |
| Meloxicam 7.5 mg x celecoxib 400 mg | - | - | - | -3.8 | (-16.6; 8.8) | Very Low*^,‡,¶^ | -3.8 | (-16.6; 8.8) | Very Low*^,‡,¶^ |
| Meloxicam 22.5 mg x meloxicam 15 mg | - | - | - | 0.4 | (-9.0; 9.8) | Low^‡,¶^ | 0.4 | (-9.0; 9.8) | Low^‡,¶^ |
| Meloxicam 22.5 mg x etoricoxib 90 mg | - | - | - | -5.2 | (-16.4; 5.9) | Very Low**^,‡,¶^ | -5.2 | (-16.4; 5.9) | Very Low**^,‡,¶^ |
| Meloxicam 22.5 mg x diclofenac 150 mg | 1.0 | (-3.3; 5.3) | Low^‡,†^ | -19.2 | (-46.9; 8.5) | Very Low^‡,†,¶^ | -1.0 | (-10.4; 8.4) | Low^‡,†^ |
| Meloxicam 22.5 mg x celecoxib 400 mg | - | - | - | -1.0 | (-13.7; 11.7) | Very Low*^,‡,¶^ | -1.0 | (-13.7; 11.7) | Very Low*^,‡,¶^ |
| Meloxicam 15 mg x etoricoxib 90 mg | - | - | - | -5.6 | (-16.8; 5.5) | Very Low**^,‡,¶^ | -5.6 | (-16.8; 5.5) | Very Low**^,‡,¶^ |
| Meloxicam 15 mg x diclofenac 150 mg | 1.4 | (-2.9; 5.7) | Moderate^‡,†^ | -18.8 | (-46.5; 8.9) | Very Low^‡,†,¶^ | -1.3 | (-10.8; 8.0) | Moderate^‡,†^ |
| Meloxicam 15 mg x celecoxib 400 mg | - | - | - | -1.0 | (-14.0; 11.3) | Very Low*^,‡,¶^ | -1.0 | (-14.0; 11.3) | Very Low*^,‡,¶^ |
| Etoricoxib 90 mg x diclofenac 150 mg | - | - | - | 4.0 | (-6.9; 15.4) | Very Low**^,‡,¶^ | 4.0 | (-6.9; 15.4) | Very Low**^,‡,¶^ |
| Etoricoxib 90 mg x celecoxib 400 mg | - | - | - | 4.0 | (-9.7; 18.3) | Very Low**^,‡,¶^ | 4.0 | (-9.7; 18.3) | Very Low**^,‡,¶^ |
| Diclofenac 150 mg x celecoxib 400mg | 2.4 | (-1.6; 6.5) | Very Low*^,‡,†^ | -20.0 | (-47.9; 7.5) | Very Low*^,‡,¶^ | 0.0 | (-8.5; 8.5) | Very Low*^,‡,†^ |

*risk of bias moderate. ** risk of bias High. ‡Imprecision. †Inconsistency. ¶ Indirectness because of questionable comparability of trial populations

CHART 6: Quality of evidence for physicians’ global assessment outcome, according to NMA GRADE

| **Comparison** | **Direct evidence** | | | **Indirect evidence** | | | **Network meta-analysis** | | |
| --- | --- | --- | --- | --- | --- | --- | --- | --- | --- |
|  | **SMD** | **95% confidence interval** | **Quality of evidence** | **SMD** | **95% confidence interval** | **Quality of evidence** | **SMD** | **95% confidence interval** | **Quality of evidence** |
| Placebo *v* naproxen 1,000 mg | - | - | - | -5.6 | (-7.4; -3.8) | Very Low**^,¶^ | -5.6 | (-7.4; -3.8) | Very Low** |
| Placebo *v* meloxicam 7.5 mg | 5.0 | (1.9; 8.0) | Moderate^†^ | -2.5 | (-30.2; 25.2) | Very Low^‡,†,¶^ | -5.0 | (-9.8; -0.1) | Moderate^†^ |
| Placebo *v* meloxicam 22.5 mg | 6.0 | (2.9; 9.0) | Moderate^†^ | -1.5 | (-29.2; 26.2) | Very Low^‡,†,¶^ | -6.0 | (-10.8; -1.1) | Moderate^†^ |
| Placebo *v* meloxicam 15 mg | 5.7 | (2.6; 8.7) | Moderate^†^ | -2.0 | (-29.4; 26.0) | Very Low^‡,†,¶^ | -6.0 | (-10.6; -0.9) | Moderate^†^ |
| Placebo *v* etoricoxib 90 mg | 9.8 | (7.7; 11.8) | Low** | 12.0 | (8.2; 15.6) | Very Low**^,‡,¶^ | -10.0 | (-12.1; -7.9) | Low** |
| Placebo *v* diclofenac 150 mg | 6.0 | (2.6; 8.8) | High | 2.0 | (-17.6; 21.6) | Low^‡,¶^ | -6.0 | (-10.6; -0.9) | **High** |
| Placebo *v* celecoxib 400 mg | - | - | - | -4.0 | (-9.6; 2.1) | Very Low*^,‡,¶^ | -4.0 | (-9.6; 2.1) | Very Low*^,‡^ |
| Naproxen 1,000 mg *v* meloxicam 7.5 mg | - | - | - | 1.0 | (-4.6; 5.8) | Very Low**^,‡,¶^ | 1.0 | (-4.6; 5.8) | Very Low**^,‡^ |
| Naproxen 1,000 mg *v* meloxicam 22.5 mg | - | - | - | -4.0 | (-5.6; 4.8) | Very Low**^,‡,¶^ | -4.0 | (-5.6; 4.8) | Very Low**^,‡^ |
| Naproxen 1,000 mg *v* meloxicam 15 mg | - | - | - | 0.0 | (-5.3; 5.0) | Very Low**^,‡,¶^ | 0.0 | (-5.3; 5.0) | Very Low**^,‡^ |
| Naproxen 1,000 mg *v* etoricoxib 90 mg | 4.9 | (2.5; 7.3) | Low** | 2.8 | (-0.6; 6.2) | Very Low**^,‡,¶^ | -4.0 | (-6.8; -2.0) | Low** |
| Naproxen 1,000 mg *v* diclofenac 150 mg | - | - | - | 0.0 | (-5.3; 5.0) | Very Low**^,‡,¶^ | 0.0 | (-5.3; 5.0) | Very Low**^,‡^ |
| Naproxen 1,000 mg *v* celecoxib 400 mg | - | - | - | 2.0 | (-4.3; 8.0) | Very Low**^,‡,¶^ | 2.0 | (-4.3; 8.0) | Very Low**^,‡^ |
| Meloxicam 7.5 mg *v* meloxicam 22.5 mg | - | - | - | -1.0 | (-5.8; 3.8) | Low^‡,¶^ | -1.0 | (-5.8; 3.8) | Low^‡^ |
| Meloxicam 7.5 mg *v* meloxicam 15 mg | - | - | - | -1.0 | (-5.6; 4.1) | Low^‡,¶^ | -1.0 | (-5.6; 4.1) | Low^‡^ |
| Meloxicam 7.5 mg *v* etoricoxib 90 mg | - | - | - | -5.0 | (-10.3; 0.3) | Very Low**^,‡,¶^ | -5.0 | (-10.3; 0.3) | Very Low |
| Meloxicam 7.5 mg *v* diclofenac 150 mg | 0.7 | (-2.3; 3.7) | Low^‡,†^ | -6.7 | (-34.4; 21.0) | Very Low^‡,†,¶^ | -1.0 | (-5.6; 4.1) | Low^‡,†^ |
| Meloxicam 7.5 mg *v* celecoxib 400 mg | - | - | - | 1.0 | (-4.6; 7.1) | Very Low*^,‡,¶^ | 1.0 | (-4.6; 7.1) | Very Low*^,‡^ |
| Meloxicam 22.5 mg *v* meloxicam 15 mg | - | - | - | 0.0 | (-4.6; 5.1) | Low^‡,¶^ | 0.0 | (-4.6; 5.1) | Low^‡^ |
| Meloxicam 22.5 mg *v* etoricoxib 90 mg | - | - | - | -4.0 | (9.3; 1.3) | Very Low**^,‡,¶^ | -4.0 | (9.3; 1.3) | Very Low**^,‡^ |
| Meloxicam 22.5 mg *v* diclofenac 150 mg | 0.2 | (-3.3; 2.9) | Moderate^‡^ | -7.7 | (-35.4; 10.0) | Low^‡,¶^ | 0.0 | (-4.6; 5.1) | Moderate^‡^ |
| Meloxicam 22.5 mg *v* celecoxib 400 mg | - | - | - | 2.0 | (-3.6; 8.1) | Very Low*^,‡,¶^ | 2.0 | (-3.6; 8.1) | Very Low*^,‡^ |
| Meloxicam 15 mg *v* etoricoxib 90 mg | - | - | - | -4.3 | (-9.6; 1.0) | Very Low**^,‡,¶^ | -4.3 | (-9.6; 1.0) | Very Low**^,‡^ |
| Meloxicam 15 mg x diclofenac 150 mg | 1.2 | (-4.3; 1.9) | Moderate^‡^ | -7.5 | (-27.1; 12.1) | Low^‡,¶^ | 0.0 | (-4.8; 4.8) | Moderate^‡^ |
| Meloxicam 15 mg x celecoxib 400 mg | - | - | - | 2.0 | (-3.9; 7.9) | Very Low*^,‡,¶^ | 2.0 | (-3.9; 7.9) | Very Low*^,‡^ |
| Etoricoxib 90 mg x diclofenac 150 mg | - | - | - | 4.0 | (-1.0; 9.6) | Very Low**^,‡,¶^ | 4.0 | (-1.0; 9.6) | Very Low**^,‡^ |
| Etoricoxib 90 mg x celecoxib 400 mg | - | - | - | 6.0 | (0.0; 12.5) | Very Low**^,‡,¶^ | 6.0 | (0.0; 12.5) | Very Low**^,‡^ |
| Diclofenac 150 mg x celecoxib 400mg | -2 | (4.5; 0.5) | Moderate* | -9.4 | (-37.1; 18.3) | Very Low*^,¶^ | 2.0 | (-1.4; 5.4) | Moderate* |

*risk of bias moderate. ** risk of bias High. ‡Imprecision. †Inconsistency. ¶ Indirectness because of questionable comparability of trial populations

CHART 7: Quality of evidence for safety outcome, according to NMA GRADE

| **Comparison** | **Direct evidence** | | | **Indirect evidence** | | | **Network meta-analysis** | | |
| --- | --- | --- | --- | --- | --- | --- | --- | --- | --- |
|  | **RR** | **95% confidence interval** | **Quality of evidence** | **RR** | **95% confidence interval** | **Quality of evidence** | **RR** | **95% confidence interval** | **Quality of evidence** |
| Placebo *v* tenoxicam 20 mg | - | - | - | - | - | - | 3.0 | 0.2; 93.4 | - |
| Placebo *v* naproxen 750 mg | - | - | - | - | - | - | 1.8 | 0.1; 29.9 | - |
| Placebo *v* naproxen 1,000 mg | 0.4 | -1; 0.6 | Very Low**^,‡,†^ | -4.0 | -1;1 | Very Low**^,‡,†,¶^ | 2.5 | 0.2; 51.6 | Very Low**^,‡,†^ |
| Placebo *v* naproxen 500 mg | - | - | - | - | - | - | 1.1 | 0.5; 2.6 | - |
| Placebo *v* nabumetone 2,000 mg | - | - | - | - | - | - | 0.9 | 0.1; 4.7 | - |
| Placebo *v* nabumetone 1,000 mg | - | - | - | - | - | - | 2.8 | 0.3; 26.7 | - |
| Placebo *v* meloxicam 7.5mg | 1.0 | -2; 2 | Low^‡,†^ | -10.0 | -10; 3 | Very Low^‡,†,¶^ | 1.4 | 0.2; 9.3 | Low^‡,†^ |
| Placebo *v* meloxicam 22.5mg | 1.0 | -2; 2 | Low^‡,†^ | -10.5 | -10.5; 2.9 | Very Low^‡,†,¶^ | 1.8 | 0.2; 12.0 | Low^‡,†^ |
| Placebo *v* meloxicam 15mg | 1.0 | -2.1; 1.8 | Low^‡,†^ | -4.8 | -4.8; 1 | Very Low^‡,†,¶^ | 2.0 | 0.4; 10.3 | Low^‡,†^ |
| Placebo *v* ketoprofen 20 mg | - | - | - | - | - | - | 0.9 | 0.1; 7.1 | - |
| Placebo *v* indomethacin 100 mg | - | - | - | - | - | - | 3.1 | 0.1; 96.6 | - |
| Placebo *v* etoricoxib 90 mg | 0.7 | -2.9; 0.1 | Very Low**^,‡,†^ | -5.3 | -5.3; 1.4 | Very Low**^,‡,†,¶^ | 4.4 | 1.2; 16.0 | Very Low**^,‡,†^ |
| Placebo *v* etodolac 50 mg | - | - | - | - | - | - | 1.9 | 0.2; 15.3 | - |
| Placebo *v* etodolac 200 mg | - | - | - | - | - | - | 1.6 | 0.2; 13.1 | - |
| Placebo *v* etodolac 100 mg | - | - | - | - | - | - | 1.9 | 0.2; 16.0 | - |
| Placebo *v* diclofenac 150 mg | 1.0 | -2.2; 1.7 | Low^‡,†^ | -5.5 | -5.5; 1.4 | Very Low^‡,†,¶^ | 1.7 | 0.2; 11.9 | Low^‡,†^ |
| Placebo *v* diclofenac 100 mg | 0 | - | - | - | - | - | 4.8 | 0.5; 46.0 | - |
| Placebo *v* celecoxib 800 mg | 1.1 | -2; 2 | Very Low**^,‡,†^ | -2.1 | -2.1; 2.4 | Very Low**^,‡,†,¶^ | 1.0 | 0.1; 6.3 | Very Low**^,‡,†^ |
| Placebo *v* celecoxib 400 mg | 1.1 | -2.6; 1.7 | Very Low**^,‡,†^ | -2.3 | -2.4; 2.0 | Very Low**^,‡,†,¶^ | 1.3 | 0.2; 8.2 | Very Low**^,‡,†^ |
| Placebo *v* celecoxib 200 mg | 1.0 | -1.9; 2.2 | Very Low**^,‡,†^ | -1.4 | -1.4; 2.3 | Very Low**^,‡,†,¶^ | 0.7 | 0.1; 3.5 | Very Low**^,‡,†^ |
| Placebo *v* aspirin 3,900 mg | - | - | - | - | - | - | 6.0 | 0.7; 50.6 | - |
| Placebo*v* aspirin 3,600 mg | - | - | - | - | - | - | 6.7 | 0.3; 139.5 | - |
| Placebo *v* aceclofenac 200 mg | - | - | - | - | - | - | 1.7 | 0.1; 27.0 | - |
| Tenoxicam 20 mg *v* naproxen 750 mg | - | - | - | - | - | - | 0.6 | 0.0; 34.1 | - |
| Tenoxicam 20 mg *v* naproxen 1,000 mg | - | - | - | - | - | - | 0.3 | 0.0; 12.8 | - |
| Tenoxicam 20 mg *v* naproxen 500 mg | - | - | - | - | - | - | 0.8 | 0.0; 63.8 | - |
| Tenoxicam 20 mg *v* nabumetone 2,000 mg | - | - | - | - | - | - | 0.3 | 0.0; 13.3 | - |
| Tenoxicam 20 mg *v* nabumetone 1,000 mg | - | - | - | - | - | - | 0.9 | 0.0; 43.1 | - |
| Tenoxicam 20 mg *v* meloxicam 7.5mg | - | - | - | - | - | - | 0.4 | 0.0; 14.8 | - |
| Tenoxicam 20 mg *v* meloxicam 22.5mg | - | - | - | - | - | - | 0.5 | 0.0; 19.1 | - |
| Tenoxicam 20 mg *v* meloxicam 15mg | - | - | - | - | - | - | 0.6 | 0.0; 20.4 | - |
| Tenoxicam 20 mg *v* ketoprofen 20 mg | - | - | - | - | - | - | 0.3 | 0.0; 16.9 | - |
| Tenoxicam 20 mg *v* indomethacin 100 mg | - | - | - | - | - | - | 1.0 | 0.0; 18.2 | - |
| Tenoxicam 20 mg *v* etoricoxib 90 mg | - | - | - | - | - | - | 1.4 | 0.0; 56.4 | - |
| Tenoxicam 20 mg *v* etodolac 50 mg | - | - | - | - | - | - | 0.6 | 0.0; 34.7 | - |
| Tenoxicam 20 mg *v* etodolac 200 mg | - | - | - | - | - | - | 0.5 | 0.0; 29.5 | - |
| Tenoxicam 20 mg *v* etodolac 100 mg | - | - | - | - | - | - | 0.6 | 0.0; 36.2 | - |
| Tenoxicam 20 mg *v* diclofenac 150 mg | - | - | - | - | - | - | 0.5 | 0.0; 10.1 | - |
| Tenoxicam 20 mg *v* diclofenac 100 mg | - | - | - | - | - | - | 1.5 | 0.0; 74.5 | - |
| Tenoxicam 20 mg *v* celecoxib 800 mg | - | - | - | - | - | - | 0.3 | 0.0; 15.4 | - |
| Tenoxicam 20 mg *v* celecoxib 400 mg | - | - | - | - | - | - | 0.4 | 0.0; 20.3 | - |
| Tenoxicam 20 mg *v* celecoxib 200 mg | - | - | - | - | - | - | 0.2 | 0.0; 8.9 | - |
| Tenoxicam 20 mg *v* aspirin 3,900 mg | - | - | - | - | - | - | 1.9 | 0.0; 112.1 | - |
| Tenoxicam 20 mg *v* aspirin 3,600 mg | - | - | - | - | - | - | 2.2 | 0.0; 171.9 | - |
| Tenoxicam 20 mg *v* aceclofenac 200 mg | - | - | - | - | - | - | 0.5 | 0.0; 4.2 | - |
| Naproxen 750 mg *v* naproxen 1,000 mg | - | - | - | - | - | - | 1.3 | 0.0; 63.5 | - |
| Naproxen 750 mg *v* naproxen 500 mg | - | - | - | - | - | - | 0.6 | 0.0; 11.1 | - |
| Naproxen 750 mg *v* nabumetone 2,000 mg | - | - | - | - | - | - | 0.5 | 0.0; 12.2 | - |
| Naproxen 750 mg *v* nabumetone 1,000 mg | - | - | - | - | - | - | 1.5 | 0.0; 40.0 | - |
| Naproxen 750 mg *v* meloxicam 7.5 mg | - | - | - | - | - | - | 0.7 | 0.1; 5.6 | - |
| Naproxen 750 mg *v* meloxicam 22.5 mg | - | - | - | - | - | - | 0.9 | 0.0; 16.4 | - |
| Naproxen 750 mg *v* meloxicam 15 mg | - | - | - | - | - | - | 1.0 | 0.0; 17.4 | - |
| Naproxen 750 mg *v* ketoprofen 20 mg | - | - | - | - | - | - | 0.5 | 0.0; 15.9 | - |
| Naproxen 750 mg *v* indomethacin 100 mg | - | - | - | - | - | - | 1.6 | 0.0; 93.8 | - |
| Naproxen 750 mg *v* etoricoxib 90 mg | - | - | - | - | - | - | 2.3 | 0.1; 50.5 | - |
| Naproxen 750 mg *v* etodolac 50 mg | - | - | - | - | - | - | 1.0 | 0.0; 33.0 | - |
| Naproxen 750 mg *v* etodolac 200 mg | - | - | - | - | - | - | 0.8 | 0.0; 28.1 | - |
| Naproxen 750 mg *v* etodolac 100 mg | - | - | - | - | - | - | 1.0 | 0.0; 34.5 | - |
| Naproxen 750 mg *v* diclofenac 150 mg | - | - | - | - | - | - | 0.9 | 0.0; 16.2 | - |
| Naproxen 750 mg *v* diclofenac 100 mg | - | - | - | - | - | - | 2.6 | 0.1; 69.0 | - |
| Naproxen 750 mg *v* celecoxib 800 mg | - | - | - | - | - | - | 0.5 | 0.0; 14.2 | - |
| Naproxen 750 mg *v* celecoxib 400 mg | - | - | - | - | - | - | 0.7 | 0.0; 18.8 | - |
| Naproxen 750 mg *v* celecoxib 200 mg | - | - | - | - | - | - | 0.3 | 0.0; 7.9 | - |
| Naproxen 750 mg *v* aspirin 3,900 mg | - | - | - | - | - | - | 3.2 | 0.1; 107.1 | - |
| Naproxen 750 mg *v* aspirin 3,600 mg | - | - | - | - | - | - | 3.6 | 0.0; 171.4 | - |
| Naproxen 750 mg *v* aceclofenac 200 mg | - | - | - | - | - | - | 0.9 | 0.0; 29.4 | - |
| Naproxen 500 mg *v* naproxen 1,000 mg | - | - | - | - | - | - | 0.4 | 0.0; 9.8 | - |
| Naproxen 500 mg *v* nabumetone 2,000 mg | - | - | - | - | - | - | 0.3 | 0.0; 10.6 | - |
| Naproxen 500 mg *v* nabumetone 1,000 mg | - | - | - | - | - | - | 1.1 | 0.1; 8.0 | - |
| Naproxen 500 mg *v* meloxicam 7.5 mg | - | - | - | - | - | - | 0.5 | 0.0; 14.3 | - |
| Naproxen 500 mg *v* meloxicam 22.5 mg | - | - | - | - | - | - | 0.7 | 0.0; 18.4 | - |
| Naproxen 500 mg *v* meloxicam 15mg | - | - | - | - | - | - | 0.7 | 0.0; 12.6 | - |
| Naproxen 500 mg *v* ketoprofen 20 mg | - | - | - | - | - | - | 0.3 | 0.0; 14.1 | - |
| Naproxen 500 mg *v* indomethacin 100 mg | - | - | - | - | - | - | 1.2 | 0.0; 93.8 | - |
| Naproxen 500 mg *v* etoricoxib 90 mg | - | - | - | - | - | - | 1.7 | 0.0; 44.9 | - |
| Naproxen 500 mg *v* etodolac 50 mg | - | - | - | - | - | - | 0.7 | 0.0; 29.2 | - |
| Naproxen 500 mg *v* etodolac 200 mg | - | - | - | - | - | - | 0.6 | 0.0; 24.9 | - |
| Naproxen 500 mg *v* etodolac 100 mg | - | - | - | - | - | - | 0.7 | 0.0; 30.5 | - |
| Naproxen 500 mg *v* diclofenac 150 mg | - | - | - | - | - | - | 0.7 | 0.0; 18.2 | - |
| Naproxen 500 mg *v* diclofenac 100 mg | - | - | - | - | - | - | 1.9 | 0.1; 32.0 | - |
| Naproxen 500 mg *v* celecoxib 800 mg | - | - | - | - | - | - | 0.4 | 0.0; 11.2 | - |
| Naproxen 500 mg *v* celecoxib 400 mg | - | - | - | - | - | - | 0.5 | 0.0; 14.8 | - |
| Naproxen 500 mg *v* celecoxib 200 mg | - | - | - | - | - | - | 0.2 | 0.0; 5.0 | - |
| Naproxen 500 mg *v* aspirin 3,900 mg | - | - | - | - | - | - | 2.3 | 0.0; 94.5 | - |
| Naproxen 500 mg *v* aspirin 3,600 mg | - | - | - | - | - | - | 2.6 | 0.1; 45.2 | - |
| Naproxen 500 mg *v* aceclofenac 200 mg | - | - | - | - | - | - | 0.6 | 0.0; 30.8 | - |
| Naproxen 1,000 mg *v* nabumetone 2,000 mg | 0.7 | -1.2; 0.6 | Very Low**^,‡,†^ | -97.3 | -97.2; 97.8 | Very Low**^,‡,†,¶^ | 0.7 | 0.2; 3.2 | Very Low**^,‡,†^ |
| Naproxen 1,000 mg *v* nabumetone 1,000 mg | - | - | - | - | - | - | 2.4 | 0.2; 24.7 | - |
| Naproxen 1,000 mg *v* meloxicam 7.5 mg | - | - | - | - | - | - | 1.2 | 0.1; 9.2 | - |
| Naproxen 1,000 mg *v* meloxicam 22.5 mg | - | - | - | - | - | - | 1.5 | 0.2; 11.9 | - |
| Naproxen 1,000 mg *v* meloxicam 15mg | - | - | - | - | - | - | 1.7 | 0.3; 10.1 | - |
| Naproxen 1,000 mg *v* ketoprofen 20 mg | - | - | - | - | - | - | 0.8 | 0.1; 7.1 | - |
| Naproxen 1,000 mg *v* indomethacin 100 mg | - | - | - | - | - | - | 2.6 | 0.0; 89.9 | - |
| Naproxen 1,000 mg *v* etoricoxib 90 mg | 0.7 | -2.9; 0.7 | Very Low**^,‡,†^ | -2.9 | -2.9; 0.7 | Very Low**^,‡,†,¶^ | 3.8 | 1.0; 13.8 | Very Low**^,‡,†^ |
| Naproxen 1,000 mg *v* etodolac 50 mg | - | - | - | - | - | - | 1.6 | 0.1; 15.3 | - |
| Naproxen 1,000 mg *v* etodolac 200 mg | - | - | - | - | - | - | 1.3 | 0.1; 13.1 | - |
| Naproxen 1,000 mg *v* etodolac 100 mg | - | - | - | - | - | - | 1.7 | 0.1; 16.0 | - |
| Naproxen 1,000 mg *v* diclofenac 150 mg | - | - | - | - | - | - | 1.5 | 0.2; 11.7 | - |
| Naproxen 1,000 mg *v* diclofenac 100 mg | - | - | - | - | - | - | 4.1 | 0.4; 42.6 | - |
| Naproxen 1,000 mg *v* celecoxib 800 mg | 1.1 | -2.2; 2.2 | Very Low**^,‡,†^ | -1.9 | -1.9; 2.7 | Very Low**^,‡,†,¶^ | 0.8 | 0.1; 5.5 | Very Low**^,‡,†^ |
| Naproxen 1,000 mg *v* celecoxib 400 mg | 1.1 | -2.5; 1.8 | Very Low**^,‡,†^ | -2.2 | -2.2; 2.3 | Very Low**^,‡,†,¶^ | 1.1 | 0.2; 7.2 | Very Low**^,‡,†^ |
| Naproxen 1,000 mg *v* celecoxib 200 mg | 1.0 | -1.8; 2.3 | Very Low**^,‡,†^ | -1.3 | -1.3; 2.7 | Very Low**^,‡,†,¶^ | 0.6 | 0.1; 3.1 | Very Low**^,‡,†^ |
| Naproxen 1,000 mg *v* aspirin 3,900 mg | - | - | - | - | - | - | 5.1 | 0.5; 50.5 | - |
| Naproxen 1,000 mg *v* aspirin 3,600 mg | - | - | - | - | - | - | 5.8 | 0.3; 126.7 | - |
| Naproxen 1,000 mg *v* aceclofenac 200 mg | - | - | - | - | - | - | 1.4 | 0.2; 25.6 | - |
| Nabumetone 2,000 mg *v* nabumetone 1,000 mg | - | - | - | - | - | - | 3.0 | 0.2; 46.2 | - |
| Nabumetone 2,000 mg *v* meloxicam 7.5 mg | - | - | - | - | - | - | 1.5 | 0.1; 18.1 | - |
| Nabumetone 2,000 mg *v* meloxicam 22.5 mg | - | - | - | - | - | - | 1.9 | 0.1; 23.3 | - |
| Nabumetone 2,000 mg *v* meloxicam 15 mg | - | - | - | - | - | - | 2.1 | 0.2; 21.0 | - |
| Nabumetone 2,000 mg *v* ketoprofen 20 mg | - | - | - | - | - | - | 1.0 | 0.2; 13.8 | - |
| Nabumetone 2,000 mg *v* indomethacin 100 mg | - | - | - | - | - | - | 3.3 | 0.2; 149.1 | - |
| Nabumetone 2,000 mg *v* etoricoxib 90 mg | - | - | - | - | - | - | 4.8 | 0.7; 32.6 | - |
| Nabumetone 2,000 mg *v* etodolac 50 mg | - | - | - | - | - | - | 2.0 | 0.1; 29.1 | - |
| Nabumetone 2,000 mg *v* etodolac 200 mg | - | - | - | - | - | - | 1.7 | 0.1; 24.9 | - |
| Nabumetone 2,000 mg *v* etodolac 100 mg | - | - | - | - | - | - | 2.1 | 0.1; 30.5 | - |
| Nabumetone 2,000 mg *v* diclofenac 150 mg | - | - | - | - | - | - | 1.9 | 0.1; 23.1 | - |
| Nabumetone 2,000 mg *v* diclofenac 100 mg | - | - | - | - | - | - | 5.2 | 0.3; 79.8 | - |
| Nabumetone 2,000 mg *v* celecoxib 800 mg | - | - | - | - | - | - | 1.0 | 0.1; 11.2 | - |
| Nabumetone 2,000 mg *v* celecoxib 400 mg | - | - | - | - | - | - | 1.4 | 0.1; 14.7 | - |
| Nabumetone 2,000 mg *v* celecoxib 200 mg | - | - | - | - | - | - | 0.7 | 0.0; 6.8 | - |
| Nabumetone 2,000 mg *v* aspirin 3,900 mg | - | - | - | - | - | - | 6.5 | 0.4; 95.3 | - |
| Nabumetone 2,000 mg *v* aspirin 3,600 mg | - | - | - | - | - | - | 7.3 | 0.2; 217.8 | - |
| Nabumetone 2,000 mg *v* aceclofenac 200 mg | - | - | - | - | - | - | 1.8 | 0.0; 44.9 | - |
| Nabumetone 1,000 mg *v* meloxicam 7.5 mg | - | - | - | - | - | - | 0.5 | 0.0; 6.6 | - |
| Nabumetone 1,000 mg *v* meloxicam 22.5 mg | - | - | - | - | - | - | 0.6 | 0.0; 8.5 | - |
| Nabumetone 1,000 mg *v* meloxicam 15 mg | 0.9 | -1.5; 2.2 | Very Low**^,‡,†^ | -91.9 | -91.9; 90.2 | Very Low**^,‡,†,¶^ | 0.7 | 0.1; 4.9 | Very Low**^,‡,†^ |
| Nabumetone 1,000 mg *v* ketoprofen 20 mg | - | - | - | - | - | - | 0.3 | 0.0; 7.0 | - |
| Nabumetone 1,000 mg *v* indomethacin 100 mg | - | - | - | - | - | - | 1.1 | 0.0; 52.6 | - |
| Nabumetone 1,000 mg *v* etoricoxib 90 mg | - | - | - | - | - | - | 1.5 | 0.1; 20.7 | - |
| Nabumetone 1,000 mg *v* etodolac 50 mg | - | - | - | - | - | - | 0.6 | 0.0; 14.7 | - |
| Nabumetone 1,000 mg *v* etodolac 200 mg | - | - | - | - | - | - | 0.5 | 0.0; 12.5 | - |
| Nabumetone 1,000 mg *v* etodolac 100 mg | - | - | - | - | - | - | 0.7 | 0.0; 15.4 | - |
| Nabumetone 1,000 mg *v* diclofenac 150 mg | - | - | - | - | - | - | 0.6 | 0.0; 8.4 | - |
| Nabumetone 1,000 mg *v* diclofenac 100 mg | 1.0 | -2.5; 1.4 | Very Low**^,‡,†^ | -92.8 | -92.3; 89.3 | Very Low**^,‡,†,¶^ | 1.7 | 0.2; 12.8 | Very Low**^,‡,†^ |
| Nabumetone 1,000 mg *v* celecoxib 800 mg | - | - | - | - | - | - | 0.3 | 0.0; 5.3 | - |
| Nabumetone 1,000 mg *v* celecoxib 400 mg | - | - | - | - | - | - | 0.4 | 0.0; 7.0 | - |
| Nabumetone 1,000 mg *v* celecoxib 200 mg | 1.0 | -0.7; 3.4 | Very Low**^,‡,†^ | -90.9 | -90.9; 91.2 | Very Low**^,‡,†,¶^ | 0.2 | 0.0; 2.0 | Very Low**^,‡,†^ |
| Nabumetone 1,000 mg *v* aspirin 3,900 mg |  | - | - | - | - | - | 2.1 | 0.1; 48.0 | - |
| Nabumetone 1,000 mg *v* aspirin 3,600 mg | 1.0 | -2.9; 1.1 | Very Low**^,‡,†^ | -128.7 | -128.7; 129.8 | Very Low**^,‡,†,¶^ | 2.4 | 0.3; 18.1 | Very Low**^,‡,†^ |
| Nabumetone 1,000 mg *v* aceclofenac 200 mg | - | - | - | - | - | - | 0.6 | 0.0; 16.0 | - |
| Meloxicam 7.5 mg *v* meloxicam 22.5mg | 1.0 | -2.2; 1.7 | Low^‡,†^ | -178.0 | -178; 176.6 | Very Low^‡,†,¶^ | 1.2 | 0.1; 9.4 | Low^‡,†^ |
| Meloxicam 7.5 mg *v* meloxicam 15 mg | 0.9 | -2.2; 1.5 | Low^‡,†^ | -178.2 | -178.1; 176.4 | Very Low^‡,†,¶^ | 1.4 | 0.2; 9.7 | Low^‡,†^ |
| Meloxicam 7.5 mg *v* ketoprofen 20 mg | - | - | - | - | - | - | 0.7 | 0.0; 10.9 | - |
| Meloxicam 7.5 mg *v* indomethacin 100 mg | - | - | - | - | - | - | 2.2 | 0.0; 72.6 | - |
| Meloxicam 7.5 mg *v* etoricoxib 90 mg | - | - | - | - | - | - | 3.1 | 0.3; 31.3 | - |
| Meloxicam 7.5 mg *v* etodolac 50 mg | - | - | - | - | - | - | 1.3 | 0.0; 23.0 | - |
| Meloxicam 7.5 mg *v* etodolac 200 mg | - | - | - | - | - | - | 1.1 | 0.0; 19.6 | - |
| Meloxicam 7.5 mg *v* etodolac 100 mg | - | - | - | - | - | - | 1.4 | 0.0; 24.1 | - |
| Meloxicam 7.5 mg *v* diclofenac 150 mg | 1.0 | -2.2; 1.7 | Low^‡,†^ | -178 | -178; 176.6 | Very Low^‡,†,¶^ | 1.2 | 0.1; 9.2 | Low^‡,†^ |
| Meloxicam 7.5 mg *v* diclofenac 100 mg | - | - | - | - | - | - | 3.4 | 0.2; 45.9 | - |
| Meloxicam 7.5 mg *v* celecoxib 800 mg | - | - | - | - | - | - | 0.7 | 0.0; 9.4 | - |
| Meloxicam 7.5mg *v* celecoxib 400 mg | - | - | - | - | - | - | 0.9 | 0.0; 12.4 | - |
| Meloxicam 7.5 mg *v* celecoxib 200 mg | - | - | - | - | - | - | 0.5 | 0.0; 4.8 | - |
| Meloxicam 7.5 mg *v* aspirin 3,900 mg | - | - | - | - | - | - | 4.3 | 0.2; 75.2 | - |
| Meloxicam 7.5 mg *v* aspirin 3,600 mg | - | - | - | - | - | - | 4.8 | 0.1; 128.9 | - |
| Meloxicam 7.5 mg *v* aceclofenac 200 mg | - | - | - | - | - | - | 4.8 | 0.1; 128.9 | - |
| Meloxicam 22.5 mg *v* meloxicam 15 mg | 1.0 | -1.8; 2.1 | Low^‡,†^ | -10.1 | -10.1; 3.4 | Very Low^‡,†,¶^ | 1.1 | 0.1; 7.5 | Low^‡,†^ |
| Meloxicam 22.5 mg *v* ketoprofen 20 mg | - | - | - | - | - | - | 0.5 | 0.0; 8.5 | - |
| Meloxicam 22.5 mg *v* indomethacin 100 mg | - | - | - | - | - | - | 1.7 | 0.0; 56.3 | - |
| Meloxicam 22.5 mg *v* etoricoxib 90 mg | - | - | - | - | - | - | 2.4 | 0.2; 24.3 | - |
| Meloxicam 22.5 mg *v* etodolac 50 mg | - | - | - | - | - | - | 1.0 | 0.0; 17.9 | - |
| Meloxicam 22.5 mg *v* etodolac 200 mg | - | - | - | - | - | - | 0.9 | 0.0; 15.2 | - |
| Meloxicam 22.5 mg *v* etodolac 100 mg | - | - | - | - | - | - | 1.1 | 0.0; 18.7 | - |
| Meloxicam 22.5 mg *v* diclofenac 150 mg | - | - | - | - | - | - | 0.9 | 0.1; 7.2 | - |
| Meloxicam 22.5 mg *v* diclofenac 100 mg | - | - | - | - | - | - | 2.6 | 0.2; 35.6 | - |
| Meloxicam 22.5 mg *v* celecoxib 800 mg | - | - | - | - | - | - | 0.5 | 0.0; 7.3 | - |
| Meloxicam 22.5mg *v* celecoxib 400 mg | - | - | - | - | - | - | 0.7 | 0.0; 9.6 | - |
| Meloxicam 22.5 mg *v* celecoxib 200 mg | - | - | - | - | - | - | 0.4 | 0.0; 3.7 | - |
| Meloxicam 22.5 mg *v* aspirin 3,900 mg | - | - | - | - | - | - | 3.3 | 0.2; 58.3 | - |
| Meloxicam 22.5 mg *v* aspirin 3,600 mg | - | - | - | - | - | - | 3.7 | 0.1; 99.9 | - |
| Meloxicam 22.5 mg *v* aceclofenac 200 mg | - | - | - | - | - | - | 0.9 | 0.0; 15.9 | - |
| Meloxicam 15 mg *v* ketoprofen 20 mg | - | - | - | - | - | - | 0.4 | 0.0; 6.3 | - |
| Meloxicam 15 mg *v* indomethacin 100 mg | - | - | - | - | - | - | 1.5 | 0.0; 47.9 | - |
| Meloxicam 15 mg *v* etoricoxib 90 mg | - | - | - | - | - | - | 2.2 | 0.3; 17.2 | - |
| Meloxicam 15 mg *v* etodolac 50 mg | - | - | - | - | - | - | 0.9 | 0.0; 13.4 | - |
| Meloxicam 15 mg *v* etodolac 200 mg | - | - | - | - | - | - | 0.8 | 0.0; 11.4 | - |
| Meloxicam 15 mg *v* etodolac 100 mg | - | - | - | - | - | - | 0.9 | 0.0; 14.0 | - |
| Meloxicam 15 mg *v* diclofenac 150 mg | 1.0 | -2.1; 1.8 | Low^‡,†^ | -1.9 | -1.9; 5.1 | Very Low^‡,†,¶^ | 0.8 | 0.1; 5.9 | Low^‡,†^ |
| Meloxicam 15 mg *v* diclofenac 100 mg | 1.0 | -2.6; 1.4 | Very Low**^,‡,†^ | -5.8 | -5.9; 1.1 | Very Low**^,‡,†,¶^ | 2.4 | 0.3; 16.3 | Very Low**^,‡,†^ |
| Meloxicam 15 mg *v* celecoxib 800 mg | - | - | - |  |  |  | 0.5 | 0.0; 4.9 |  |
| Meloxicam 15mg *v* celecoxib 400 mg | 1.1 | -0.5; 3.9 | Very Low**^,‡,†^ | -2.7 | -2.7; 2.7 | Very Low**^,‡,†,¶^ | 0.6 | 0.0; 6.4 | Very Low**^,‡,†^ |
| Meloxicam 15 mg *v* celecoxib 200 mg | - | - | - | - | - | - | 0.3 | 0.0; 2.0 | - |
| Meloxicam 15 mg *v* aspirin 3,900 mg | - | - | - | - | - | - | 2.9 | 0.2; 43.8 | - |
| Meloxicam 15 mg *v* aspirin 3,600 mg | - | - | - | - | - | - | 3.3 | 0.2; 54.3 | - |
| Meloxicam 15 mg *v* aceclofenac 200 mg | - | - | - | - | - | - | 0.8 | 0.0; 13.4 | - |
| Ketoprofen 20 mg *v* indomethacin 100 mg | - | - | - | - | - | - | 3.1 | 0.0; 168.0 | - |
| Ketoprofen 20 mg *v* etoricoxib 90 mg | - | - | - | - | - | - | 4.5 | 0.4; 48.2 | - |
| Ketoprofen 20 mg *v* etodolac 50 mg | - | - | - | - | - | - | 1.9 | 0.1; 34.6 | - |
| Ketoprofen 20 mg *v* etodolac 200 mg | - | - | - | - | - | - | 1.6 | 0.0; 29.5 | - |
| Ketoprofen 20 mg *v* etodolac 100 mg | - | - | - | - | - | - | 2.0 | 0.1; 36.2 | - |
| Ketoprofen 20 mg *v* diclofenac 150 mg | - | - | - | - | - | - | 1.8 | 0.1; 28.3 | - |
| Ketoprofen 20 mg *v* diclofenac 100 mg | - | - | - | - | - | - | 4.9 | 0.2; 99.5 | - |
| Ketoprofen 20 mg *v* celecoxib 800 mg | - | - | - | - | - | - | 1.0 | 0.0; 15.3 | - |
| Ketoprofen 20 mg *v* celecoxib 400 mg | - | - | - | - | - | - | 1.3 | 0.0; 20.2 | - |
| Ketoprofen 20 mg *v* celecoxib 200 mg | - | - | - | - | - | - | 0.7 | 0.0; 9.3 | - |
| Ketoprofen 20 mg *v* aspirin 3,900 mg | - | - | - | - | - | - | 6.1 | 0.3; 112.9 | - |
| Ketoprofen 20 mg *v* aspirin 3,600 mg | - | - | - | - | - | - | 6.9 | 0.1; 258.0 | - |
| Ketoprofen 20 mg *v* aceclofenac 200 mg | - | - | - | - | - | - | 1.7 | 0.0; 52.2 | - |
| Indomethacin 100 mg *v* etoricoxib 90 mg | - | - | - | - | - | - | 1.4 | 0.0; 55.2 | - |
| Indomethacin 100 mg *v* etodolac 50 mg | - | - | - | - | - | - | 0.6 | 0.0; 33.9 | - |
| Indomethacin 100 mg *v* etodolac 200 mg | - | - | - | - | - | - | 0.5 | 0.0; 28.9 | - |
| Indomethacin 100 mg *v* etodolac 100 mg | - | - | - | - | - | - | 0.6 | 0.0; 35.5 | - |
| Indomethacin 100 mg *v* diclofenac 150 mg | - | - | - | - | - | - | 0.5 | 0.0; 9.9 | - |
| Indomethacin 100 mg *v* diclofenac 100 mg | - | - | - | - | - | - | 1.5 | 0.0; 72.9 | - |
| Indomethacin 100 mg *v* celecoxib 800 mg | - | - | - | - | - | - | 0.3 | 0.0; 15.0 | - |
| Indomethacin 100 mg *v* celecoxib 400 mg | - | - | - | - | - | - | 0.4 | 0.0; 19.9 | - |
| Indomethacin 100 mg *v* celecoxib 200 mg | - | - | - | - | - | - | 0.2 | 0.0; 8.7 | - |
| Indomethacin 100 mg *v* aspirin 3,900 mg | - | - | - | - | - | - | 1.9 | 0.0; 109.7 | - |
| Indomethacin 100 mg *v* aspirin 3,600 mg | - | - | - | - | - | - | 2.1 | 0.0; 168.1 | - |
| Indomethacin 100 mg *v* aceclofenac 200 mg | - | - | - | - | - | - | 0.5 | 0.0; 4.2 | - |
| Etoricoxib 90 mg *v* etodolac 50 mg | - | - | - | - | - | - | 0.4 | 0.0; 5.0 | - |
| Etoricoxib 90 mg *v* etodolac 200 mg | - | - | - | - | - | - | 0.3 | 0.0; 4.2 | - |
| Etoricoxib 90 mg *v* etodolac 100 mg | - | - | - | - | - | - | 0.4 | 0.0; 5.2 | - |
| Etoricoxib 90 mg *v* diclofenac 150 mg | - | - | - | - | - | - | 0.4 | 0.0; 3.9 | - |
| Etoricoxib 90 mg *v* diclofenac 100 mg | - | - | - | - | - | - | 1.0 | 0.0; 14.2 | - |
| Etoricoxib 90 mg *v* celecoxib 800 mg | - | - | - | - | - | - | 0.2 | 0.0; 2.0 | - |
| Etoricoxib 90 mg *v* celecoxib 400 mg | - | - | - | - | - | - | 0.3 | 0.0; 2.6 | - |
| Etoricoxib 90 mg *v* celecoxib 200 mg | - | - | - | - | - | - | 0.1 | 0.0; 1.1 | - |
| Etoricoxib 90 mg *v* aspirin 3,900 mg | - | - | - | - | - | - | 1.3 | 0.1; 16.4 | - |
| Etoricoxib 90 mg *v* aspirin 3,600 mg | - | - | - | - | - | - | 1.5 | 0.0; 40.0 | - |
| Etoricoxib 90 mg *v* aceclofenac 200 mg | - | - | - | - | - | - | 0.3 | 0.0; 8.0 | - |
| Etodolac 50 mg *v* etodolac 200 mg | - | - | - | - | - | - | 0.8 | 0.1; 6.8 | - |
| Etodolac 50 mg *v* etodolac 100 mg | - | - | - | - | - | - | 1.0 | 0.1; 8.3 | - |
| Etodolac 50 mg *v* diclofenac 150 mg | - | - | - | - | - | - | 0.9 | 0.0; 15.7 | - |
| Etodolac 50 mg *v* diclofenac 100 mg | - | - | - | - | - | - | 2.5 | 0.1; 54.7 | - |
| Etodolac 50 mg *v* celecoxib 800 mg | - | - | - | - | - | - | 0.5 | 0.0; 8.5 | - |
| Etodolac 50 mg *v* celecoxib 400 mg | - | - | - | - | - | - | 0.7 | 0.0; 11.2 | - |
| Etodolac 50 mg *v* celecoxib 200 mg | - | - | - | - | - | - | 0.3 | 0.0; 5.2 | - |
| Etodolac 50 mg *v* aspirin 3,900 mg | - | - | - | - | - | - | 3.1 | 0.3; 26.3 | - |
| Etodolac 50 mg *v* aspirin 3,600 mg | - | - | - | - | - | - | 3.5 | 0.0; 140.3 | - |
| Etodolac 50 mg *v* aceclofenac 200 mg | - | - | - | - | - | - | 0.9 | 0.0; 28.5 | - |
| Etodolac 200 mg *v* etodolac 100 mg | - | - | - | - | - | - | 1.2 | 0.1; 9.9 | - |
| Etodolac 200 mg *v* diclofenac 150 mg | - | - | - | - | - | - | 1.1 | 0.0; 18.6 | - |
| Etodolac 200 mg *v* diclofenac 100 mg | - | - | - | - | - | - | 2.9 | 0.1; 64.9 | - |
| Etodolac 200 mg *v* celecoxib 800 mg | - | - | - | - | - | - | 0.6 | 0.0; 10.1 | - |
| Etodolac 200 mg *v* celecoxib 400 mg | - | - | - | - | - | - | 0.8 | 0.0; 13.3 | - |
| Etodolac 200 mg *v* celecoxib 200 mg | - | - | - | - | - | - | 0.4 | 0.0; 6.1 | - |
| Etodolac 200 mg *v* aspirin 3,900 mg | - | - | - | - | - | - | 3.7 | 0.4; 31.3 | - |
| Etodolac 200 mg *v* aspirin 3,600 mg | - | - | - | - | - | - | 4.2 | 0.1; 166.3 | - |
| Etodolac 200 mg *v* aceclofenac 200 mg | - | - | - | - | - | - | 1.0 | 0.0; 33.8 | - |
| Etodolac 100 mg *v* diclofenac 150 mg | - | - | - | - | - | - | 0.8 | 0.0; 15.0 | - |
| Etodolac 100 mg *v* diclofenac 100 mg | - | - | - | - | - | - | 2.4 | 0.1; 52.5 | - |
| Etodolac 100 mg *v* celecoxib 800 mg | - | - | - | - | - | - | 0.5 | 0.0; 8.1 | - |
| Etodolac 100 mg *v* celecoxib 400 mg | - | - | - | - | - | - | 0.6 | 0.0; 10.7 | - |
| Etodolac 100 mg *v* celecoxib 200 mg | - | - | - | - | - | - | 0.3 | 0.0; 5.0 | - |
| Etodolac 100 mg *v* aspirin 3,900 mg | - | - | - | - | - | - | 3.0 | 0.3; 25.3 | - |
| Etodolac 100 mg *v* aspirin 3,600 mg | - | - | - | - | - | - | 3.4 | 0.0; 134.6 | - |
| Etodolac 100 mg *v* aceclofenac 200 mg | - | - | - | - | - | - | 0.8 | 0.0; 27.3 | - |
| Diclofenac 150 mg *v* diclofenac 100 mg | - | - | - | - | - | - | 2.7 | 0.2; 35.9 | - |
| Diclofenac 150 mg *v* celecoxib 800 mg | - | - | - | - | - | - | 0.5 | 0.0; 7.4 | - |
| Diclofenac 150 mg *v* celecoxib 400 mg | - | - | - | - | - | - | 0.7 | 0.0; 9.7 | - |
| Diclofenac 150 mg *v* celecoxib 200 mg | - | - | - | - | - | - | 0.4 | 0.0; 3.8 | - |
| Diclofenac 150 mg *v* aspirin 3,900 mg | - | - | - | - | - | - | 3.3 | 0.1; 59.0 | - |
| Diclofenac 150 mg *v* aspirin 3,600 mg | - | - | - | - | - | - | 3.8 | 0.1;101.1 | - |
| Diclofenac 150 mg *v* aceclofenac 200 mg | 1.0 | -1.9; 2.0 | Very Low**^,‡,†^ | -84.5 | -84.6; 86.1 | Very Low**^,‡,†,¶^ | 0.9 | 0.1; 7.1 | Very Low**^,‡,†^ |
| Diclofenac 100 mg *v* celecoxib 800 mg | - | - | - | - | - | - | 0.2 | 0.0; 3.0 | - |
| Diclofenac 100 mg *v* celecoxib 400 mg | - | - | - | - | - | - | 0.2 | 0.0; 4.0 | - |
| Diclofenac 100 mg *v* celecoxib 200 mg | - | - | - | - | - | - | 0.1 | 0.0; 1.1 | - |
| Diclofenac 100 mg *v* aspirin 3,900 mg | - | - | - | - | - | - | 1.2 | 0.0; 27.7 | - |
| Diclofenac 100 mg *v* aspirin 3,600 mg | - | - | - | - | - | - | 1.4 | 0.0; 24.0 | - |
| Diclofenac 100 mg *v* aceclofenac 200 mg | - | - | - | - | - | - | 0.3 | 0.0; 9.2 | - |
| Celecoxib 800 mg *v* celecoxib 400 mg | - | - | - | - | - | - | 1.3 | 0.1; 10.7 | - |
| Celecoxib 800 mg *v* celecoxib 200 mg | - | - | - | - | - | - | 0.7 | 0.1; 5.3 | - |
| Celecoxib 800 mg *v* aspirin 3,900 mg | - | - | - | - | - | - | 5.9 | 0.3; 99.2 | - |
| Celecoxib 800 mg *v* aspirin 3,600 mg | - | - | - | - | - | - | 6.7 | 0.2; 193.3 | - |
| Celecoxib 800 mg *v* aceclofenac 200 mg | - | - | - | - | - | - | 1.6 | 0.0; 43.8 | - |
| Celecoxib 400 mg *v* celecoxib 200 mg | - | - | - | - | - | - | 0.5 | 0.0; 3.8 | - |
| Celecoxib 400 mg *v* aspirin 3,900 mg | - | - | - | - | - | - | 4.4 | 0.2; 72.9 | - |
| Celecoxib 400 mg *v* aspirin 3,600 mg | - | - | - | - | - | - | 5.0 | 0.1; 142.3 | - |
| Celecoxib 400 mg *v* aceclofenac 200 mg | - | - | - | - | - | - | 1.2 | 0.0; 32.2 | - |
| Celecoxib 200 mg *v* aspirin 3,900 mg | - | - | - | - | - | - | 8.2 | 0.5; 117.8 | - |
| Celecoxib 200 mg *v* aspirin 3,600 mg | - | - | - | - | - | - | 9.3 | 0.5; 166.9 | - |
| Celecoxib 200 mg *v* aceclofenac 200 mg | - | - | - | - | - | - | 2.3 | 0.1; 47.4 | - |
| Aspirin 3,900 mg *v* aspirin 3,600 mg | - | - | - | - | - | - | 1.1 | 0.0; 45.7 | - |
| Aspirin 3,900 mg *v* aceclofenac 200 mg | - | - | - | - | - | - | 0.2 | 0.0; 9.3 | - |
| Aspirin 3,600 mg *v* aceclofenac 200 mg | - | - | - | - | - | - | 0.2 | 0.0; 11.6 | - |

Note: date for indirect meta-analysis could not be calculated by the statistical program..

*risk of bias moderate. ** risk of bias High. ‡Imprecision. †Inconsistency. ¶ Indirectness because of questionable comparability of trial populations
