# Supplementary material for: Use of corticoids and non-steroidal anti-inflammatories in the treatment of rheumatoid arthritis: Systematic review and network meta-analysis
Source: PLoS One. 2021 Apr 7;16(4):e0248866. doi: 10.1371/journal.pone.0248866 (PMC8026036; doi:10.1371/journal.pone.0248866)
Supplement: S4 File — (DOCX) [file pone.0248866.s005.docx]

S4 File. Reason for exclusion of studies

| **Study** | **Reason for exclusion** |
| --- | --- |
| Adler, S. "Early rheumatoid arthritis: Less joint erosion with methotrexate, prednisolone and/or cyclosporine." 33 (2008): 190-192. | Not found abstract or full text |
| Ammitzbøll, F. "Fenbufen and indomethacin in the treatment of rheumatoid arthritis. A comparative double-blind, crossover study." *Scandinavian journal of rheumatology. Supplement* 23 (1979): 5-10. | Not found abstract or full text |
| Ardia, A., et al. "Comparative Studies with Tolfenamic Acid in Rheumatic Disorders." *Pharmacology & toxicology* 75 (1994): 66-71. | Not found abstract or full text |
| Auteri, A., et al. "Effect of a long-term treatment with two different corticosteroids on patients suffering from rheumatoid arthritis: clinical and immunological study." *International journal of immunotherapy* 10.2 (1994): 67-75. | Not found abstract or full text |
| Azuma, T., et al. "Long-term comparative studies on gold, D-penicillamine, and NSAIDs for the treatment of early rheumatoid arthritis. 1. Evaluation of one year's treatment." *Ryumachi.[Rheumatism]* 26.3 (1986): 200-209. | Not found abstract or full text |
| Bickham, Kara, et al. "Evaluation of two doses of etoricoxib, a COX-2 selective non-steroidal anti-inflammatory drug (NSAID), in the treatment of Rheumatoid Arthritis in a double-blind, randomized controlled trial." BMC musculoskeletal disorders17.1 (2016): 331. | Wrong intervention |
| Blackburn Jr, Warren D., et al. "Tenidap in rheumatoid arthritis a 24‐week double‐blind comparison with hydroxychloroquine‐plus‐piroxicam, and piroxicam alone." Arthritis & Rheumatism38.10 (1995): 1447-1456. | Wrong intervention |
| Bensen, W., et al. "Efficacy and safety of valdecoxib in treating the signs and symptoms of rheumatoid arthritis: a randomized, controlled comparison with placebo and naproxen." Rheumatology 41.9 (2002): 1008-1016. | Non-commercially drug |
| Blechman, W. J., and B. L. Lechner. "Clinical comparative evaluation of choline magnesium trisalicylate and acetylsalicylic acid in rheumatoid arthritis." Rheumatology 18.2 (1979): 119-124. | Wrong intervention |
| Boers, M., et al. "What is the relationship between morning symptoms and measures of disease activity in patients with rheumatoid arthritis?." Arthritis care & research 67.9 (2015): 1202-1209. | wrong type of study |
| Bombardier, C., P. M. Peloso, and C. H. Goldsmith. "Salsalate, a nonacetylated salicylate, is as efficacious as diclofenac in patients with rheumatoid arthritis. Salsalate-Diclofenac Study Group." The Journal of rheumatology 22.4 (1995): 617-624. | Not found abstract or full text |
| Briancon, D. "International experience with etodolac therapy for rheumatoid arthritis: an interim report of comparative efficacy." Clinical rheumatology 8.1 (1989): 63-72. | wrong population of study |
| Buttgereit, Frank, et al. "Targeting pathophysiological rhythms: prednisone chronotherapy shows sustained efficacy in rheumatoid arthritis." *Annals of the rheumatic diseases* 69.7 (2010): 1275-1280. | Wrong intervention |
| Buttgereit, Frank, et al. "Efficacy of modified-release versus standard prednisone to reduce duration of morning stiffness of the joints in rheumatoid arthritis (CAPRA-1): a double-blind, randomised controlled trial." *The Lancet* 371.9608 (2008): 205-214. | Wrong intervention |
| Cardoe, N., and F. Dudley Hart. "Double‐blind multicentre UK hospital studies of isoxicam vs naproxen." *British journal of clinical pharmacology* 22.S2 (1986): 167S-172S. | Wrong intervention |
| Caruso, I., et al. "Lornoxicam versus diclofenac in rheumatoid-arthritis: a double-blind, multicenter study." *Advances in Therapy* 11.3 (1994): 132-138. | Not found abstract or full text |
| Ciompi, M. L., et al. "Etodolac versus diclofenac: double-blind cross-over study in rheumatoid arthritis." *International journal of clinical pharmacology research* 9.3 (1989): 217-222. | Wrong type of study |
| De, I. Salcedo. "Fenbufen--a new nonsteroidal anti-inflammatory agent: comparison with phenylbutazone in rheumatoid arthritis." *Current therapeutic research, clinical and experimental* 18.2 (1975): 295-302. | Not found abstract or full text |
| Eichler, H-G., et al. "Association between health-related quality of life and clinical efficacy endpoints in rheumatoid arthritis patients after four weeks treatment with anti-inflammatory agents." *International Journal of Clinical Pharmacology & Therapeutics* 43.5 (2005). | Not found abstract or full text |
| Emery, P., et al. "Nabumetone compared with naproxen in the treatment of rheumatoid arthritis: a multicenter, double blind, randomized, parallel group trial in hospital outpatients." *The Journal of rheumatology. Supplement* 36 (1992): 41-47. | Not found abstract or full text |
| Furst, Daniel E., et al. "A controlled study of concurrent therapy with a nonacetylated salicylate and naproxen in rheumatoid arthritis." *Arthritis & Rheumatism: Official Journal of the American College of Rheumatology* 30.2 (1987): 146-154. | Wrong type of study |
| Goekoop‐Ruiterman, YPM D., et al. "Clinical and radiographic outcomes of four different treatment strategies in patients with early rheumatoid arthritis (the BeSt study): a randomized, controlled trial." *Arthritis & Rheumatism* 52.11 (2005): 3381-3390. | Wrong intervention |
| Goekoop-Ruiterman, Yvonne PM, et al. "Comparison of treatment strategies in early rheumatoid arthritis: a randomized trial." *Annals of internal medicine* 146.6 (2007): 406-415. | Wrong intervention |
| Havranek, H. "Double-blind study of tenoxicam 20 mg versus piroxicam 20 mg in rheumatoid arthritis." *European journal of rheumatology and inflammation* 9.2 (1987): 105. | Not found abstract or full text |
| Hill, J., et al. "A double‐blind crossover study to compare lysine acetyl salicylate (aspergesic) with ibuprofen in the treatment of rheumatoid arthritis." *Journal of clinical pharmacy and therapeutics* 15.3 (1990): 205-211. | Wrong type of study |
| Imbimbo, B., et al. "Clinical equivalence of a new glucocorticoid, deflazacort and prednisone in rheumatoid arthritis and SLE patients." *Advances in experimental medicine and biology* 171 (1984): 241. | Not found abstract or full text |
| Jonderko, G., et al. "Evaluation of the efficacy and tolerability of nabumetone and piroxicam in patients with reumatoid arthritis." *REUMATOLOGIA-WARSAW-* 36 (1998): 49-55. | Not found abstract or full text |
| Kahabbazi, A., et al. "Comparing control of rheumatoid arthritis flare up in pulse therapy with dexamethasone and methylprednisolone: 703274." *International Journal of Rheumatic Diseases* 15 (2012). | Not found abstract or full text |
| Kellner, Herbert L., Chunming Li, and Margaret N. Essex. "Efficacy and safety of celecoxib versus diclofenac and omeprazole in elderly arthritis patients: a subgroup analysis of the CONDOR trial." Current medical research and opinion 28.9 (2012): 1537-1545. | Wrong population of study |
| Kellner, Herbert L., Chunming Li, and Margaret N. Essex. "Celecoxib and diclofenac plus omeprazole are similarly effective in the treatment of arthritis in patients at high GI risk in the CONDOR trial." *The open rheumatology journal* 7 (2013): 96. | Wrong population of study |
| Kessler, S., et al. "The Role of Intraarticular Glucocorticoid Injections for the Outcome after 3 Months in Polyarticular Active Rheumatoid Arthritis." *Aktuelle Rheumatologie* 34.06 (2009): 356-362. | Not found abstract or full text |
| Kessler, S., et al. "The Role of Intraarticular Glucocorticoid Injections for the Outcome after 3 Months in Polyarticular Active Rheumatoid Arthritis." *Aktuelle Rheumatologie* 34.06 (2009): 356-362. | Not found abstract or full text |
| Laine, L., et al. "Risk factors for NSAID‐associated upper GI clinical events in a long‐term prospective study of 34 701 arthritis patients." *Alimentary pharmacology & therapeutics*32.10 (2010): 1240-1248. | Wrong outcome |
| Lemmel, E. M., et al. "Efficacy and safety of meloxicam in patients with rheumatoid arthritis." *The Journal of rheumatology* 24.2 (1997): 282-290. | Not found abstract or full text |
| Lisse, Jeffrey R. "Clinical efficacy and safety of Naprelan versus Naprosyn in the treatment of rheumatoid arthritis." *American journal of orthopedics (Belle Mead, NJ)* 25.9 Suppl (1996): 21-29. | Not found abstract or full text |
| Lipsky, P. E., and P. C. Isakson. "Outcome of specific COX-2 inhibition in rheumatoid arthritis." *The Journal of rheumatology. Supplement* 49 (1997): 9-14. | Not found abstract or full text |
| Lucca, F., M. G. Souto, and J. R. Silva. "Comparative study of 2 corticosteroids, in a test of double anonymity, in the treatment of the rheumatoid arthritis." *Hospital (Rio de Janeiro, Brazil)* 70.4 (1966): 981-990. | Not found abstract or full text |
| Malaia, L. T., M. M. Liashenko, and VIa Brigidina. "Dynamics of the joint pains at night in rheumatoid arthritis and arthroses treated with Rengasil and piroxicam." *Farmakologiia i toksikologiia* 49.6 (1986): 83-87. | Not found abstract or full text |
| Marcos, F. Sánchez, et al. "Proglumetacin in the treatment of rheumatoid arthritis." *Anales de medicina interna (Madrid, Spain: 1984)*. Vol. 6. No. 4. 1989. | Not found abstract or full text |
| Markusse, Iris M., et al. "Long-term outcomes of patients with recent-onset rheumatoid arthritis after 10 years of tight controlled treatment: a randomized trial." *Annals of internal medicine* 164.8 (2016): 523-531. | Wrong intervention |
| Masi, A. T., and R. T. Chatterton. "Glucocorticoid-like anti-inflammatory versus immunosuppressive effects of CPH 82 as a single drug therapy of moderately active rheumatoid arthritis patients." *Scandinavian journal of rheumatology* 29.2 (2000): 85-88. | Not found abstract or full text |
| Neustadt, David H. "Double blind evaluation of the long-term effects of etodolac versus ibuprofen in patients with rheumatoid arthritis." *The Journal of rheumatology. Supplement* 47 (1997): 17-22. | Not found abstract or full text |
| Orozco-Alcalá, J. J., and E. F. Barrera-Tenorio. "Long-term treatment with tenoxicam in rheumatoid arthritis." *European journal of rheumatology and inflammation* 9.2 (1987): 118-121. | Not found abstract or full text |
| Palmer, M., J. Highton, and D. G. Palmer. "A double blind comparison of tiaprofenic acid with placebo." *The New Zealand medical journal* 101.845 (1988): 240-241. | Not found abstract or full text |
| Paulus, Harold E., et al. "Patient retention and hand-wrist radiograph progression of rheumatoid arthritis during a 3-year prospective study that prohibited disease modifying antirheumatic drugs." *The Journal of rheumatology* 31.3 (2004): 470-481. | Not found abstract or full text |
| Pavelka, K., D. Kanková, and O. Vojtísĕk. "Comparison of 3 therapeutic regimes using non-steroidal antirheumatic agents in rheumatoid arthritis." *Fysiatricky a reumatologicky vestnik*59.5 (1981): 250. | Not found abstract or full text |
| Pavelka, K. and Stolfa, Jirí. "Coxibs in the treatment of osteoarthrosis and rheumatoid arthritis." 5 (2002): 89-96. | Not found abstract or full text |
| Perepel'chenko, A. I., et al. "Use of flugalin and profenid in patients with rheumatoid arthritis." *Vrachebnoe delo* 6 (1988): 38-40. | Not found abstract or full text |
| Prupas, H. M., et al. "Tenidap in Patients with Rheumatoid Arthritis." *Scandinavian journal of rheumatology* 25.6 (1996): 345-351. | Not found abstract or full text |
| Safy, Mary, et al. "Long-term outcome is better when a methotrexate-based treatment strategy is combined with 10 mg prednisone daily: follow-up after the second Computer-Assisted Management in Early Rheumatoid Arthritis trial." Annals of the rheumatic diseases 76.8 (2017): 1432-1435. | Wrong outcome |
| Schnitzer, Thomas J., et al. "The safety profile, tolerability, and effective dose range of rofecoxib in the treatment of rheumatoid arthritis." *Clinical therapeutics* 21.10 (1999): 1688-1702. | Wrong intervention |
| Shichikawa, K., I. Nagaya, and N. Ogawa. "Double-blind clinical trial of pirprofen in patients with rheumatoid arthritis." *Ryumachi.[Rheumatism]* 24.5 (1984): 407-414. | Not found abstract or full text |
| Soldati, R., G. Sorbilli, and P. Di Benedetto. "Long-term comparative study of fenbufen and aspirin in patients with rheumatoid arthritis." *La Clinica terapeutica* 92.4 (1980): 417. | Not found abstract or full text |
| Solomon, Daniel H., et al. "Differences in safety of nonsteroidal antiinflammatory drugs in patients with osteoarthritis and patients with rheumatoid arthritis: a randomized clinical trial." Arthritis & Rheumatology 70.4 (2018): 537-546. | Wrong population of study |
| ten Wolde, Saskia, et al. "Randomised placebo-controlled study of stopping second-line drugs in rheumatoid arthritis." *The Lancet* 347.8998 (1996): 347-352. | Wrong type of study |
| Van Jaarsveld, C. H. M., et al. "Toxicity of anti‐rheumatic drugs in a randomized clinical trial of early rheumatoid arthritis." *Rheumatology* 39.12 (2000): 1374-1382. | Wrong intervention |
| Verschueren, P., et al. "Methotrexate in combination with other DMARDs is not superior to methotrexate alone for remission induction with moderate-to-high-dose glucocorticoid bridging in early rheumatoid arthritis after 16 weeks of treatment: the CareRA trial." *Annals of the rheumatic diseases* 74.1 (2015): 27-34. | Wrong intervention |
| Verstappen, Suzan MM, et al. "Five‐year followup of rheumatoid arthritis patients after early treatment with disease‐modifying antirheumatic drugs versus treatment according to the pyramid approach in the first year." *Arthritis & Rheumatism: Official Journal of the American College of Rheumatology* 48.7 (2003): 1797-1807. | Wrong intervention |
| Wankya, B. M. "Tolfenamic acid and ibuprofen in rheumatoid arthritis: a double-blind cross over study." *East Afr Med J* 58.8 (1981): 622-625. | Not found abstract or full text |
