# Supplementary material for: Use of corticoids and non-steroidal anti-inflammatories in the treatment of rheumatoid arthritis: Systematic review and network meta-analysis
Source: PLoS One. 2021 Apr 7;16(4):e0248866. doi: 10.1371/journal.pone.0248866 (PMC8026036; doi:10.1371/journal.pone.0248866)
Supplement: S5 File — (DOCX) [file pone.0248866.s006.docx]

S5 File. Studies included in the review.

**Nonsteroidal anti-inflammatories**

BERNHARD, G. C. et al. Long-term treatment of rheumatoid arthritis comparing nabumetone with aspirin. **The American journal of medicine,** v. 83, n. 4, p. 44-49, 1987. ISSN 0002-9343.

COLLANTES, E. et al. A multinational randomized, controlled, clinical trial of etoricoxib in the treatment of rheumatoid arthritis [ISRCTN25142273]. **BMC Family Practice,** v. 3, n. 1, p. 10, 2002. ISSN 1471-2296.

EMERY, P. et al. Nabumetone compared with naproxen in the treatment of rheumatoid arthritis: a multicenter, double blind, randomized, parallel group trial in hospital outpatients. **The Journal of rheumatology. Supplement,** v. 36, p. 41-47, 1992. ISSN 0380-0903.

EMERY, P. et al. Celecoxib versus diclofenac in long-term management of rheumatoid arthritis: randomised double-blind comparison. **The Lancet,** v. 354, n. 9196, p. 2106-2111, 1999. ISSN 0140-6736.

FURST, D. E. et al. Dose response and safety study of meloxicam up to 22.5 mg daily in rheumatoid arthritis: a 12 week multicenter, double blind, dose response study versus placebo and diclofenac. **The Journal of rheumatology,** v. 29, n. 3, p. 436-446, 2002. ISSN 0315-162X.

GEUSENS, P. et al. Efficacy, safety and tolerability of lumiracoxib in patients with rheumatoid arthritis. **International journal of clinical practice,** v. 58, n. 11, p. 1033-1041, 2004. ISSN 1368-5031.

GEUSENS, P. et al. A placebo and active comparator-controlled trial of rofecoxib for the treatment of rheumatoid arthritis. **Scandinavian journal of rheumatology,** v. 31, n. 4, p. 230-238, 2002. ISSN 0300-9742.

GIBOFSKY, A. et al. Efficacy and tolerability of valdecoxib in treating the signs and symptoms of severe rheumatoid arthritis: a 12-week, multicenter, randomized, double-blind, placebo-controlled study. **Clinical therapeutics,** v. 29, n. 6, p. 1071-1085, 2007. ISSN 0149-2918.

JACOB, G. et al. Minimum effective dose of etodolac for the treatment of rheumatoid arthritis. **The Journal of Clinical Pharmacology,** v. 26, n. 3, p. 195-202, 1986. ISSN 0091-2700.

KAWAI, S. et al. Efficacy and Safety of Ketoprofen Patch in Patients With Rheumatoid Arthritis: A Randomized, Double‐Blind, Placebo‐Controlled Study. **The Journal of Clinical Pharmacology,** v. 50, n. 10, p. 1171-1179, 2010. ISSN 0091-2700.

KORNASOFF, D. et al. The efficacy and tolerability of aceclofenac compared to indomethacin in patients with rheumatoid arthritis. **Rheumatology international,** v. 15, n. 6, p. 225-230, 1996. ISSN 0172-8172.

KRUG, H. et al. Tolerability and efficacy of nabumetone and naproxen in the treatment of rheumatoid arthritis. **Clinical therapeutics,** v. 22, n. 1, p. 40-52, 2000. ISSN 0149-2918.

LIGHTFOOT, R. Comparison of the efficacy and safety of etodolac and piroxicam in patients with rheumatoid arthritis. Etodolac Study 326 Rheumatoid Arthritis Investigators Group. **The Journal of rheumatology. Supplement,** v. 47, p. 10-16, 1997. ISSN 0380-0903.

MATSUMOTO, A. K. et al. A randomized, controlled, clinical trial of etoricoxib in the treatment of rheumatoid arthritis. **The Journal of rheumatology,** v. 29, n. 8, p. 1623-1630, 2002. ISSN 0315-162X.

PASERO, G. et al. A multi-centre, double-blind comparative study of the efficacy and safety of aceclofenac and diclofenar in the treatment of rheumatoid arthritis. **Current medical research and opinion,** v. 13, n. 6, p. 305-315, 1995. ISSN 0300-7995.

PEREZ-RUIZ, F.; ALONSO-RUIZ, A.; ANSOLEAGA, J. Comparative study of the efficacy and safety of aceclofenac and tenoxicam in rheumatoid arthritis. **Clinical rheumatology,** v. 15, n. 5, p. 473-477, 1996. ISSN 0770-3198.

SHI, W. et al. Safety and efficacy of oral nonsteroidal anti-inflammatory drugs in patients with rheumatoid arthritis. **Clinical drug investigation,** v. 24, n. 2, p. 89-101, 2004. ISSN 1173-2563.

VASEY, F. B. et al. Controlled evaluation of nabumetone in the treatment of active adult rheumatoid arthritis: nabumetone versus naproxen double-blind parallel study. **The American journal of medicine,** v. 83, n. 4, p. 55-59, 1987. ISSN 0002-9343.

WILLIAMS, G. W. et al. A comparison of valdecoxib and naproxen in the treatment of rheumatoid arthritis symptoms. **Clinical therapeutics,** v. 28, n. 2, p. 204-221, 2006. ISSN 0149-2918.

WOJTULEWSKI, J. et al. A six-month double-blind trial to compare the efficacy and safety of meloxicam 7.5 mg daily and naproxen 750 mg daily in patients with rheumatoid arthritis. **Rheumatology,** v. 35, n. suppl_1, p. 22-28, 1996. ISSN 1462-0332.

ZHAO, S. Z. et al. Evaluation of health‐related quality of life of rheumatoid arthritis patients treated with celecoxib. **Arthritis Care & Research,** v. 13, n. 2, p. 112-121, 2000. ISSN 0004-3591.

**Steroidal anti-inflammatories**

BAKKER, M. F. et al. Low-dose prednisone inclusion in a methotrexate-based, tight control strategy for early rheumatoid arthritis: a randomized trial. **Ann Intern Med,** v. 156, n. 5, p. 329-39, Mar 6 2012. ISSN 0003-4819.

BUTTGEREIT, F. et al. Low-dose prednisone chronotherapy for rheumatoid arthritis: a randomised clinical trial (CAPRA-2). **Ann Rheum Dis,** v. 72, n. 2, p. 204-10, Feb 2013. ISSN 0003-4967.

CHOY, E. H. et al. Factorial randomised controlled trial of glucocorticoids and combination disease modifying drugs in early rheumatoid arthritis. **Ann Rheum Dis,** v. 67, n. 5, p. 656-63, May 2008. ISSN 0003-4967.

DING, C. Z. et al. Clinical analysis of chinese patients with rheumatoid arthritis treated with leflunomide and methotrexate combined with different dosages of glucocorticoid. **Curr Ther Res Clin Exp,** v. 73, n. 4-5, p. 123-33, Sep 2012. ISSN 0011-393X (Print) 0011-393x.

HAFSTROM, I. et al. Rheumatoid factor and anti-CCP do not predict progressive joint damage in patients with early rheumatoid arthritis treated with prednisolone: a randomised study. **BMJ Open,** v. 4, n. 7, p. e005246, Jul 30 2014. ISSN 2044-6055 (Print) 2044-6055.
