# Supplementary material for: Use of corticoids and non-steroidal anti-inflammatories in the treatment of rheumatoid arthritis: Systematic review and network meta-analysis
Source: PLoS One. 2021 Apr 7;16(4):e0248866. doi: 10.1371/journal.pone.0248866 (PMC8026036; doi:10.1371/journal.pone.0248866)
Supplement: S6 File — (DOCX) [file pone.0248866.s007.docx]

S6 File. Heterogeneity of studies according to analyzed outcomes.

**Pain**

**Physical function**

**Number of tender/painful joints and swollen joints**

^^

**Patients’ and physicians’ global assessment**

**Safety of the interventions**
